# Enamelin Is Critical for Ameloblast Integrity and Enamel Ultrastructure Formation

**DOI:** 10.1371/journal.pone.0089303

**Published:** 2014-03-06

**Authors:** Jan C.-C. Hu, Yuanyuan Hu, Yuhe Lu, Charles E. Smith, Rangsiyakorn Lertlam, John Timothy Wright, Cynthia Suggs, Marc D. McKee, Elia Beniash, M. Enamul Kabir, James P. Simmer

**Affiliations:** 1 Dental Research Laboratory, University of Michigan School of Dentistry, Ann Arbor, Michigan, United States of America; 2 Facility for Electron Microscopy Research, Department of Anatomy and Cell Biology, and Faculty of Dentistry, McGill University, Montreal, QC, Canada; 3 Dental Research Center, University of North Carolina at Chapel Hill, Chapel Hill, North Carolina, United States of America; 4 McGill University, Faculty of Dentistry, and Department of Anatomy and Cell Biology, Montreal, QC, Canada; 5 Department of Oral Biology, School of Dental Medicine, University of Pittsburgh, Pittsburgh, Pennsylvania, United States of America; University of Massachusetts Medical, United States of America

## Abstract

Mutations in the human enamelin gene cause autosomal dominant hypoplastic *amelogenesis imperfecta* in which the affected enamel is thin or absent. Study of enamelin knockout NLS-*lacZ* knockin mice revealed that mineralization along the distal membrane of ameloblast is deficient, resulting in no true enamel formation. To determine the function of enamelin during enamel formation, we characterized the developing teeth of the *Enam^−/−^* mice, generated amelogenin-driven enamelin transgenic mouse models, and then introduced enamelin transgenes into the *Enam^−/−^* mice to rescue enamel defects. Mice at specific stages of development were subjected to morphologic and structural analysis using β-galactosidase staining, immunohistochemistry, and transmission and scanning electron microscopy. Enamelin expression was ameloblast-specific. In the absence of enamelin, ameloblasts pathology became evident at the onset of the secretory stage. Although the aggregated ameloblasts generated matrix-containing amelogenin, they were not able to create a well-defined enamel space or produce normal enamel crystals. When enamelin is present at half of the normal quantity, enamel was thinner with enamel rods not as tightly arranged as in wild type suggesting that a specific quantity of enamelin is critical for normal enamel formation. Enamelin dosage effect was further demonstrated in transgenic mouse lines over expressing enamelin. Introducing enamelin transgene at various expression levels into the *Enam^−/−^* background did not fully recover enamel formation while a medium expresser in the *Enam^+/−^* background did. Too much or too little enamelin abolishes the production of enamel crystals and prism structure. Enamelin is essential for ameloblast integrity and enamel formation.

## Introduction

Dental enamel, which has the hardness of mild steel [1], is formed by ameloblasts. Ameloblasts are a tooth-specific cell type, that secrete three major enamel-specific matrix proteins: amelogenin, ameloblastin, and enamelin. These matrix proteins are expressed by secretory stage ameloblasts and are critical for normal enamel biomineralization. After expanding the enamel matrix to its final dimensions, secretory stage ameloblasts undergo a transition to maturation stage that alters their expression of matrix proteins. During the maturation stage, degradation and reabsorption of matrix proteins becomes a priority of ameloblasts, because such events allow space to be freed up for enamel crystals to increase in width and thickness, leading to the hardening of dental enamel. Mature dental enamel contains less than 1% of organic matter [2]. Based on studies of gene-targeted mouse models, absence of amelogenin [3], ameloblastin [4] or enamelin [5], results in significantly decreased enamel thickness and altered prism structure.

In humans, enamelin gene mutations result in autosomal dominant hypoplastic *amelogenesis imperfecta (OMIM 104500 AIH2 and 204650 AI1C)*. Individuals with both *ENAM* alleles defective typically present with severe enamel hypoplasia, while individuals with one allele mutated have localized, pitted defects and/or horizontal grooves on their teeth [6], [7]. Enamelin was first isolated from developing porcine enamel matrix [8]. Full length enamelin is a large glycoprotein having apparent molecular weight ∼186 kDa [9]. In the developing enamel, rapid cleavage of enamelin mediated by matrix metalloproteinase 20 (MMP20) takes place immediately upon secretion. The C-terminus of enamelin concentrates near the mineralization front where crystal initiation and elongation takes place, while the major cleavage product, 32 kDa enamelin, distributes throughout the developing enamel layer and accumulates to make up about 1% of the total enamel matrix [10]. Compared to amelogenin, the major extracellular constituent comprising ∼90% of the developing enamel matrix, enamelin and its cleavage products are present at a lesser quantity, only about 5% of the total enamel matrix. The importance of enamelin in dental enamel formation has been demonstrated unequivocally by a chemically induced mouse model [11], gene-targeted mouse model [5] and human mutations giving rise to hypoplastic *amelogenesis imperfecta* [12]. The consistent observation of abnormally thin and disorganized enamel in *Enam* null mice and in human AI suggests that enamelin function is required to direct crystal formation and to achieve prism structural organization, and optimal enamel thickness. Loss of tooth or enamel formation during evolution is associated with molecular decay of the enamelin gene [13].

The abnormal enamel development of *Enam* ^+/−^ and *Enam* ^−/−^ mouse teeth was illustrated by morphological analyses of the enamelin (*Enam* ID:13801) knockout *lacZ* knockin mouse model [5]. Based on the observation of more than 10 generations of the *Enam* ^−/−^ breeding colony, enamelin absence affected litter size, weight gain and survival of the young pups if the *Enam* ^−/−^ mice were not maintained on soft diet [14]. Furthermore, because the abnormally thin enamel altered inter-dental contacts, secondary effects of molar drifting were observed in the *Enam* ^−/−^ mice, such as decreased bone volume between the first and second molars [15]. This observation indicated that when the space distal to the first molar was open due to the absence of enamel, the teeth responded by shifting to close the space. However, bone density was not altered among the *Enam* ^−/−^ mice, which suggested that the observed changes in periodontal bone level were secondary to molar drifting and super-eruption caused by the lack of occlusal and interdental contacts due to enamel hypoplasia and attrition. At the histological level, the most striking effect of enamelin absence was the lack of mineral formation at the mineralization front that ultimately resulted in no mineralization of the enamel layer as demonstrated by von Kossa staining of non-demineralized PN7 first molar sections [5]. In the *Enam* ^−/−^ mice, premature and excessive apoptosis of ameloblasts contributed to overall cellular disorganization and enamel hypoplasia [16].

In this study, our objective was to define the enamel ultrastructural defects of the enamelin knockout lacZ knockin mouse and to determine the effect of enamelin transgene expression in the knockout background. Experiments were conducted to characterize the cellular and structural anomalies of enamel occurring in the *Enam* ^+/−^ and *Enam* ^−/−^ mouse teeth. Transgenic mice over expressing enamelin at minimal, medium and high levels were established, characterized and bred with the *Enam* ^−/−^ mice separately. The effect of transgenic enamelin on enamel formation in the *Enam* ^−/−^ background was assessed morphologically. We hypothesized that if the amount of enamelin comparable to that of the wild type could be introduced into the *Enam* ^−/−^ background, then the defective enamel structure and thickness should be restored.

## Materials and Methods

### Protocol approval

Animal protocols were reviewed and approved by the University of Michigan Institutional Animal Care and Use Committee.

### Beta-galactosidase staining

Beta-galactosidase whole-mount staining of embryonic day 13.5, newborn, and postnatal day 7 (PN7) and day 14 (PN14) mice from wild type, enamelin knockin heterozygous (*Enam* ^+/−^) and homozygous (*Enam* ^−/−^) mice were processed with the removal of intestines. Skin was removed from samples for genotyping. Experiments were done in triplicate. Separately, internal organs from PN7 *Enam* ^−/−^ mice were processed for cryosectioning and β-gal staining. Day 14 mice were perfused via the heart with 4% paraformaldehyde (PFA) and 0.1% glutaraldehyde (GA) and all other samples were collected following conventional protocol. Half maxillae were collected and fixed overnight, washed with phosphate-buffered saline (PBS, 3×15 min), then decalcified in 10% ethylenediamine tetraacetic acid (EDTA) up to 7 days according to the age of the mice. The PN7 decalcified tissues were washed in PBS (3×15 min) and embedded in Tissue-Tek O.C.T. Compound (ProSciTech, Queensland, Australia). Whole mouse samples and PN7 tissue blocks sectioned at 10 µm thickness were post-fixed for 5 min in 0.5% GA, washed in 100 mM, pH 8.0 Hepes buffer (3×5 min), and incubated at 45°C for 2 h in freshly prepared X-gal staining buffer, pH 8.0, containing 1 mg/ml X-gal, 100 mM Hepes, 5 mM potassium ferricyanide, 1 mM MgCl_2_, 2% Triton X-100. Tissue sections were rinsed and stored in 100 mM, pH 8.0 Hepes buffer for counterstaining with hematoxylin then observed under a dissection microscope (Nikon SMZ1000) or light microscope (Nikon Eclipse E600). All images were captured using a digital camera (Nikon Digital Sight) and NIS-Elements basic research imaging software V4.10 (Mager Scientific, Dexter, MI, USA).

### Immunohistochemistry

The techniques used for immunohistochemistry were described previously (Simmer et al., 2009). PN7 mouse heads were dissected and fixed by immersion in ice cold 4% PFA and 1% GA in a 67 mM phosphate buffer, pH 7.4 overnight. PN14 mice were perfused with ice cold 4% PFA and 1% GA in a 67 mM phosphate buffer, pH 7.4. Mouse maxillae and mandibles were demineralized using 10% EDTA for 5 to 7 days. The fixed maxillary and mandibular processes were dissected, embedded and sectioned at 5 µm. The slides of the maxillary molars were deparaffinized 3× in xylene, rehydrated and treated with peroxidase blocking agent. The sections were incubated with serum blocking solution and then incubated with amelogenin antibodies (rm179 at 1∶2,000) at room temperature for 30 min and enamelin antibodies (mENAM^223–236^ at 1∶100) at 4°C for overnight. Negative control slides were incubated with pre-immune serum at the same concentrations as the primary antibodies. Following incubation with primary antibodies (or pre-immune serum in the negative control), the slides were washed 3× with PBS, incubated with biotinylated secondary antibody, washed 3×, incubated with streptavidin-enzyme conjugate, washed 3×, incubated with substrate-chromogen mixture, washed 3×, counterstained with hematoxylin, and mounted with Aqueous Mounting Solution (Invitrogen, Carlsbad, CA) for microscopy and photography.

### SEM analyses

Ethanol dehydrated, air-dried, fractured mandibular incisors from 7-week-old wild type, *Enam* ^+/−^, *Enam* ^−/−^ and transgenic mice were mounted with conductive carbon cement onto metallic stubs, de-gassed in a vacuum desiccator overnight, sputter coated with Au-Pd thin film to increase conductivity, and imaged at Amray EF 1910 Scanning Electron Microscope operating at an accelerating voltage of 3–5 kV. For backscattered SEM, samples were polished with silicone carbide paper (South Bay Technology, Inc. San Clemente, CA) followed by 4 h of polishing with 1.0 micro alumina abrasive with Multitex Polishing Cloth using a Buehler Supermet 2 Position Polisher (Lake Bluff, IL), then sonicated and rinsed with water. The finely polished tooth surface was coated with carbon and imaged using the Cameca SX-100 Electron Microprobe Analyzer (Cameca, Gennevilliers Cedex, FR) at the University of Michigan Electron Microbeam Analysis Laboratory (EMAL) using the backscatter mode at a beam current of 15 kV and 10 nA.

### TEM evaluation

Transmission electron microscopy (TEM) was used to determine ameloblast and enamel morphology and to investigate the secretion and localization of amelogenin. Briefly, one-week-old mandibles of wild type, *Enam* ^+/−^ and *Enam* ^−/−^ mice were fixed in 4% PFA plus 1.0% GA in 0.1 M sodium cacodylate buffer, pH 7.3. Mandibles were left undecalcified for embedding in Epon epoxy resin (Cedarlane, Burlington, ON), or were decalcified for immunogold labeling in 8% EDTA over 2 weeks followed by embedding in LR White acrylic plastic (London Resin Company, Berkshire, UK). Samples destined for embedding in Epon for morphological analysis were additionally osmicated for 1 h in potassium ferrocyanide-reduced 1% osmium tetroxide. Prior to embedding, all samples were dehydrated in a graded ethanol series, infiltrated with the embedding media, placed into mounting molds and the blocks were polymerized at 55°C for 2 days. Thin sections (80 nm) were cut using a Leica Ultracut E ultramicrotome (Leica, Wetzlar, Germany) followed by staining with uranyl acetate and lead citrate after which the sections were viewed in a FEI Technai 12 transmission electron microscope (Hillsboro, OR) operating at 120 kV and equipped with a 792 Bioscan 1 k×1 k wide-angle multiscan CCD camera (Pleasanton, CA). For immunogold labeling of amelogenin prior to TEM evaluation, LR White sections were incubated with polyclonal rabbit anti-porcine 25 kDa amelogenin (Dr. T. Uchida, Hiroshima University, Japan) followed by protein A-colloidal gold (14 nm) conjugate (Dr. G. Posthuma, University of Utrecht, Utrecht, The Netherlands) [17].

### Generation of enamelin transgenic founders and breeding with *Enam* ^−/−^ mice

Six PCR primers were designed to amplify target sequences and to introduce rare (8 base cutter) restriction sites (Fig. S1). The *AmelX* promoter (5′*AmelX*, 4655 bp), the *Enam* cDNA (*Enam*, 3845 bp) and *AmelX* downstream (3′*AmelX*, 1143 bp) sequence were amplified, subcloned into pCR2.1-TOPO (3931 bp) and sequence confirmed. Plasmids having the 5′ ends of the PCR products on the *Not*I side of the vector were used to construct the *Enam* transgene. The plasmid containing *AmelX* promoter sequence (4.6 kb) and *Enam* cDNA sequence (3.8 kb) were restricted with *Asc*I and *Not*I, ligated and sequenced. The *AmelX* promoter-*Enam* cDNA fusion to *AmelX* 3′ sequence involved *Srf*I and *Sgf*I restriction (Fig. S2). Following DNA sequencing confirmation (Fig. S3), the correct construct of 13.5 kb was restricted with *Not*I-*Srf*I to yield the 9.6 kb *Enam* transgene with *Amelx* regulatory sequences, which was microinjected into fertilized C57BL/6 X SJL F2 oocytes and surgically transferred to recipients at the Transgenic Animal Model Core at the University of Michigan. A total of 13 independent lines were generated and bred with C57BL/6 mice. Germline transmission was determined by PCR analyses of genomic DNA obtained from tail biopsies of the offspring. The transgenic lines designated as line 2, 3, 7, 11, and 12 were propagated and their offspring characterized. Offspring carrying the *Enam* transgene (*Enam* ^+/+,tg^) were mated with *Enam* ^−/−^ mice. Two breeding strategies were used. *Enam* ^+/+,tg^ mice were used to breed with C57/BL6 wild type mice to generate transgenic mice (*Enam* ^+/+,tg^) or wild type mice (*Enam* ^+/+^); *Enam* ^+/+,tg^ mice were used to breed with *Enam* ^−/−^ mice to generate F1 offspring. *Enam* transgene positive F1 (*Enam* ^+/−,tg^) were used to breed with *Enam* null mice again to generate F2 offspring. Four genotypes were generated: *Enam* ^−/−,tg^, *Enam* ^+/−,tg^, *Enam* ^−/−^ and *Enam* ^+/−^. Genotyping was carried out using the three primer sets, *Enam* tg, *Enam* 4&5, and *lac*Z. (Fig. S4).

### Assessment of *Enam* transgene expression levels by ELISA

Day 5 molars from the right mandibles and maxillae of mice with specific genotypes were extracted and incubated on a rotator with 1 mL of HF solution (0.17 N HCl + 0.95 N formic acid) that contained both the protease inhibitors (Cocktail Set-III-EDTA free, EMD Millipore Corporation, Billerica, MA) and phenanthroline at 1 mM concentrations for 3 h at 4°C. Samples were centrifuged (10000 rpm, 3 min) to remove residual insoluble material, and then the supernatant was neutralized with 6 N NaOH with a final pH ∼5. Desalting and buffer exchange was carried out by using 0.01% formic acid and Amicon 4 mL ultracentrifuge concentrators (molecular weight cutoff = 3 kDa; EMD) according to manufacturer instructions. After desalting, sample volume was raised to 1 mL using 0.01% formic acid and frozen at −80°C for more than 1 h, then lyophilized overnight. Lyophilized samples were re-dissolved in 250 µL 0.1 M bicarbonate buffer (pH 9.6) containing protease inhibitors.

To determine the quantity of enamelin, samples were diluted to the desired concentration, 1/50^th^ of the total protein, in 50 µL of coating buffer (0.1 M sodium bicarbonate, pH 9.6) and coated onto the wells of 96-well microplates (Immulon 2 HB, Fisher Scientific, Pittsburgh, PA), incubated overnight at 4°C, washed three times with PBS containing 0.01% Tween 20 (PBS-T) and then blocked for 1 h at room temperature with 4% skimmed milk in PBS-T. The plates were then washed three times with PBS-T and 50 µL of mENAM^223–236^ antibody (1∶1,000 dilution in 4% skimmed milk in PBS-T) was added and incubated for 2 h at room temperature. The plates were washed three times with PBS-T, incubated with 50 µL (5000-fold diluted in 4% skimmed milk in PBS-T) of HRP-conjugated anti-rabbit IgG antibody from donkey (GE Healthcare, Pittsburgh, PA) for 1 h at room temperature. The wells were washed three times with PBS-T before 50 µL of 0.022% of 2′, 2′-azino-bis-3-ethylbenzthiazoline-6-sulfonic acid in citric acid with 0.05% hydrogen peroxide was added to each well for 15 min, and then the absorbance was measured at 405 nm on a Microplate Reader 680 (BioRad, Hercules, CA). Glyceraldehyde-3-phosphate dehydrogenase (GAPDH) was used as internal control for normalization. Equal quantity of protein, 1/50^th^ of the total protein of two Day 5 teeth, in 50 µL coating buffer (0.1 M sodium bicarbonate, pH 9.6) was used in the ELISA assay. Rabbit polyclonal anti-GAPDH antibody with a 1∶500 dilution in 4% skimmed milk was used as a primary antibody and HRP-conjugated anti-rabbit IgG antibody at 1∶5000 from donkey (GE Healthcare) was used as secondary antibody to detect the GAPDH.

### Von Kossa staining

Day 4 mandibles of offspring from wild type, line 2 and line 3 Tg mice were fixed by immersion in 4% PFA/0.1% GA, dehydrated in increasing concentrations of ethanol, embedded in paraffin and sectioned at 5 µm. Sections were selected, de-paraffinized and rehydrated. Von Kossa staining for mineral was performed by applying 1% silver nitrate in distilled water to the sections and exposing them to ultraviolet light wavelength 302 nm for 20 min. Unreacted silver was removed with 5% sodium thiosulfate for 5 min [18]. Sections were washed 3 times in distilled water, counterstained with methyl green for 3 min, rinsed in distilled water, dehydrated through graded ethanol, cleared in xylene and coverslips were mounted using Permount (Fisher Scientific, Fair Lawn, NJ) for microscopy and photography.

## Results

### Enamelin expression determined by *lac*Z histostaining

**Figure 1:**
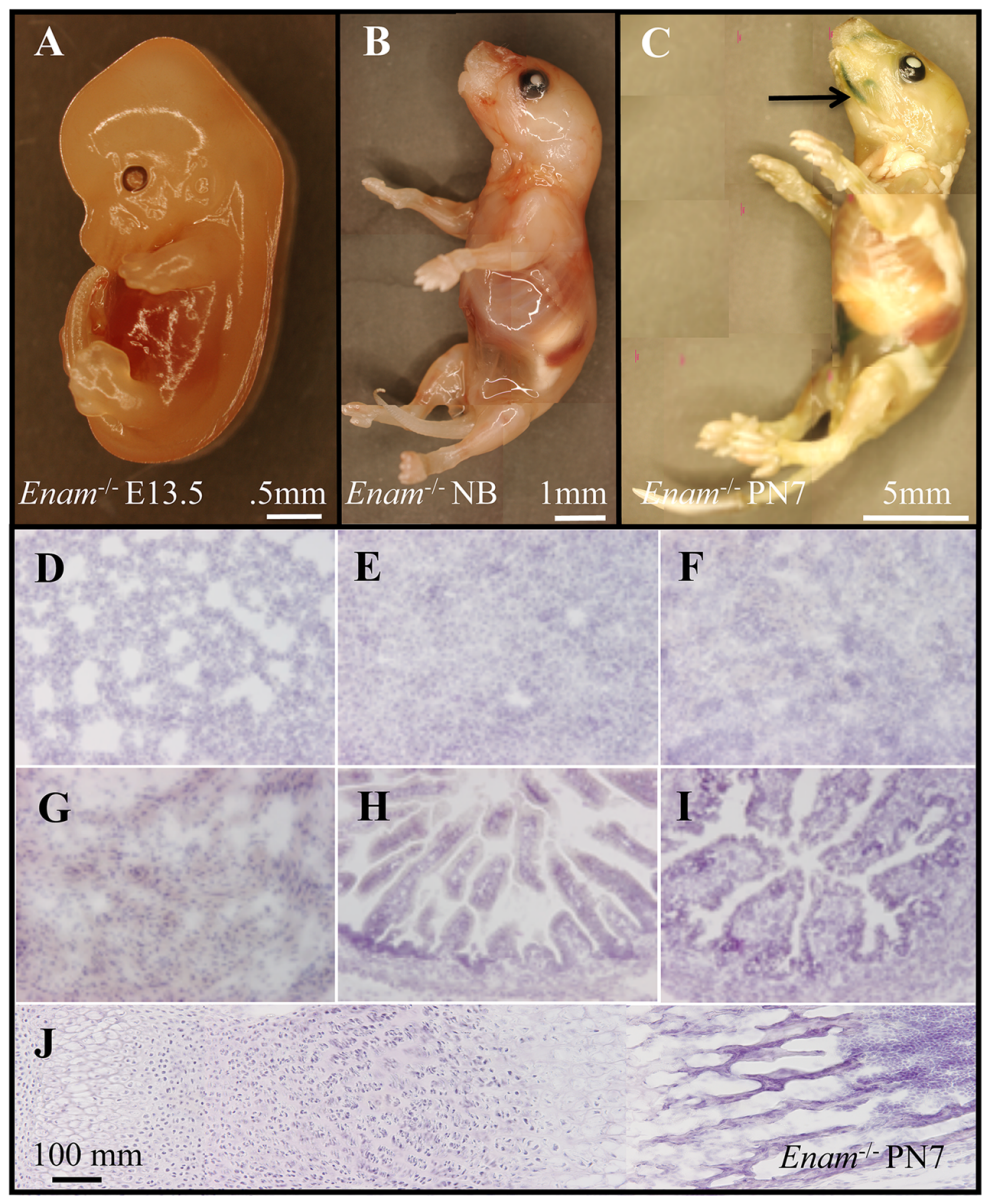
Enamelin expression depicted by β-galactosidase whole-mount staining of *Enam* knockout *lacZ* knockin mice. (A) Embryonic day 13.5, (B) newborn, and (C) PN7 mice were processed with and without the removal of intestines. Separately, internal organs from PN7 *Enam* ^−/−^ mice were processed for cryosectioning and β-gal staining; no positive staining was observed in (D) lung, (E) liver, (F) kidney, (G) stomach, (H) small and (I) large intestines, or (J) cartilage from long bones.

**Figure 2:**
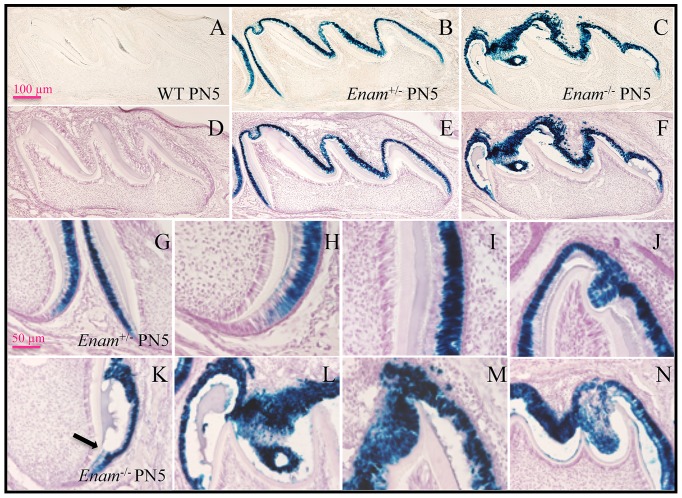
Beta-galactosidase staining of maxillary molars from PN5 wild type, *Enam* ^+/−^ and *Enam* ^−/−^ mice. (A, unstained; D, H&E counter stained) No positive staining is consistently observed in wild type samples. (B, E) Positive staining is observed in the *Enam* ^+/−^ molars localized to the well-polarized ameloblast layer outlining the developing enamel space and showed no detectable differences from wild type molars in terms of ameloblast organization, cell height and thickness of the enamel layer. (C, F) In the *Enam* ^−/−^ molars, abnormal accumulations of enamel matrix and changes in ameloblast morphology and alignment were evident on PN5. (K) Ameloblasts lost polarity and were unable to maintain an even enamel space soon after enamel formation would have normally begun (arrow). (L–N) Irregular aggregation of ameloblasts and extracellular matrix material is apparent.

**Figure 3:**
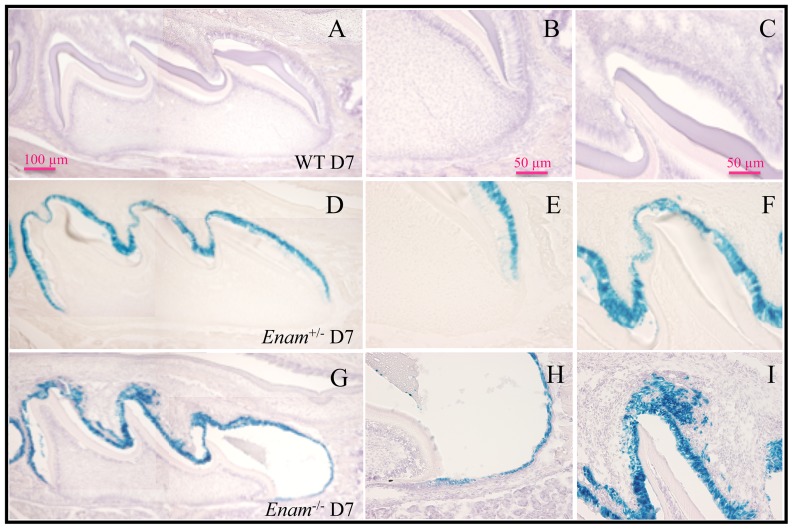
Beta-galactosidase staining of maxillary molars from PN7 collected from wild type, *Enam* ^+/−^, and *Enam* ^−/−^ mice. (A–C, H&E counter stained) No positive staining is consistently observed in wild type samples. (D–F, unstained) There are no detectable differences between wild type and *Enam* ^+/−^ molars in terms of ameloblast organization, size of the ameloblasts and thickness of the enamel layer. (G–I) In *Enam* ^−/−^ molars, bulges of enamel matrix along the cuspal slopes are associated with flattening ameloblasts. Non-polarized, *lac*Z positive cells are seen instead of ameloblasts and extending into the stratum intermedium area, sometimes incorporated in the abnormal aggregation of matrix and cellular components near the cusp tips in the null mouse samples.

**Figure 4:**
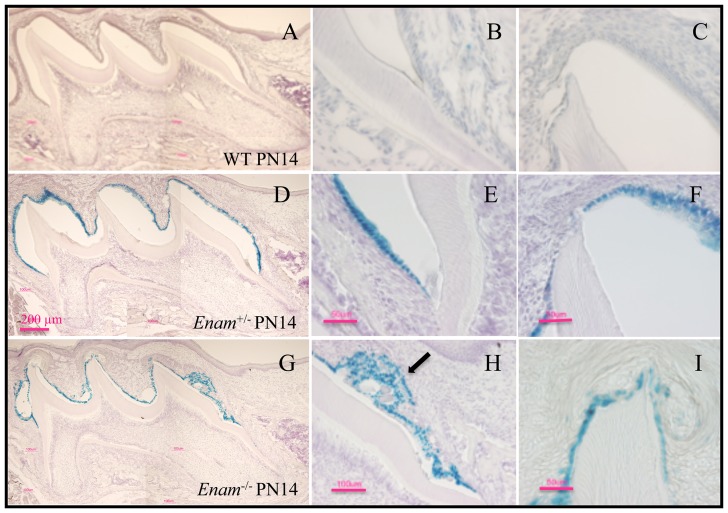
Beta-galactosidase staining of maxillary molars from PN14 collected from wild type, *Enam* ^+/−^, and *Enam* ^−/−^ mice. (A–C) No positive staining is observed in wild type samples. (D–F) There are no appreciable abnormalities in *Enam* ^+/−^ molars. In *Enam* ^−/−^ molars, abnormal aggregations are present along the cuspal slopes (arrow). (G–I) The enamel organ is highly disorganized compared to expected appearance for normal maturation stage and the dentin is covered by an abnormally thin disorganized matrix.

The enamelin KO/*lacZ* KI construct replaced the *Enam* gene segment from the start codon in exon 3 to the 5′ end of intron 7, keeping the entire 5′ regulatory region inclusive of exon 1/intron 1, exon2/intron 2, and the 5′ end of exon 3 intact [5]. Enamelin temporal and spatial expression pattern was characterized using β-gal staining of embryonic day 13.5, newborn and postnatal day 7 (PN7) *Enam* ^−/−^ mice. *LacZ* positive staining was observed in developing incisors and molars from day 7 and on. Although greenish blue stain was initially observed in the intestine, when the intestinal contents were washed clean, no further positive staining occurred. Histologically, no positive staining for enamelin expression was detected in major organs of PN7 mice, which included lung, liver, kidney, stomach, small intestines, large intestine and cartilage of the long bone (Fig. 1). *Lac*Z-positive staining in the histological sections of the developing teeth from PN 5 (Fig. 2), 7 (Fig. 3), and 14 (Fig. 4) *Enam* ^+/−^ and *Enam* ^−/−^ mice was specifically associated with ameloblasts while there was no positive staining among the comparable wild type negative control samples. During the secretory stage, normal enamel matrix and ameloblasts were seen in *Enam* ^+/−^ molars (Fig. 2G–J), while abnormal accumulation of enamel matrix proteins and detachment of ameloblasts were apparent in all null mouse teeth (Fig. 2K–N). Although some irregularity of the ameloblast cell layers, specifically homogeneity and continuity, existed among *Enam* ^+/−^ molars at all time points, there was no apparent variation of enamel thickness of these teeth. The pathologic changes in the developing *Enam* ^−/−^ molars increased at PN7 during which time ameloblasts completely lost their polarity and aggregated abnormally (Fig. 3G–I). The enamel space was irregularly thin except for a significant bulge was often observed on the mesial cuspal slope. At PN14, the *Enam* ^−/−^ molars failed to achieve the normal enamel thickness (Fig. 4G). The ameloblasts lacked a Tomes' process and tall columnar shape, but retained *lac*Z expression and deposited matrix and amorphous calcifications along the molar mesial and distal cusp slopes (Fig. 4H).

### Enamelin expression determined by IHC

**Figure 5:**
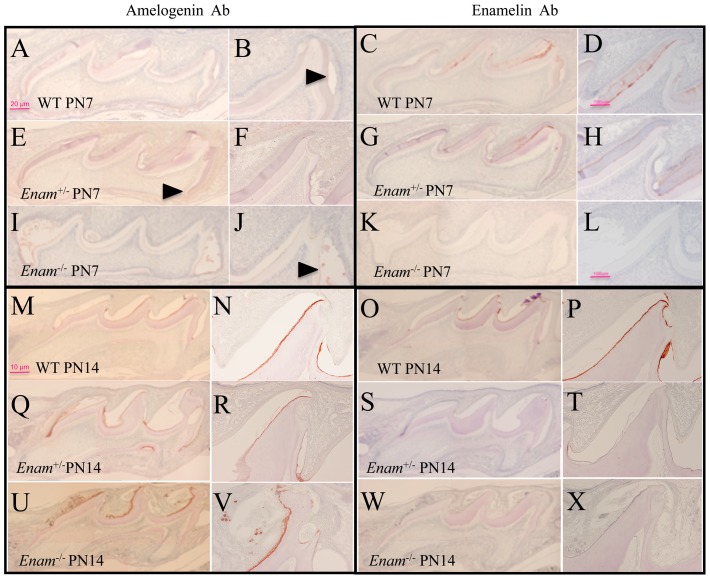
Immunohistochemical staining of PN7 and PN14 molars using amelogenin rm179 polyclonal and enamelin mENAM^223–236^ antipeptide antibodies. (A–V) Weak amelogenin positive staining can be detected in PN7 and PN14 molars of wild type (A–B and M–N, arrowhead), *Enam* ^+/−^ (E–F and Q–R, arrowhead), and *Enam* ^−/−^ (I–J and U–V, arrowhead) mice. (C–D versus G–H) A similar distribution pattern but with slightly different intensities of enamelin staining is apparent comparing wild type to *Enam* ^+/−^ samples. The enamelin positive staining is evenly distributed across the entire thickness of enamel layer in wild type and *Enam* ^+/−^ samples. (K–L) However, no positive staining can be detected in *Enam* ^−/−^ molars. (I, K, U, W) Abnormal accumulations of organic matrix are evident on the mesial and distal cusp slopes in *Enam* ^−/−^ samples. (M–V) The trend continued into PN14, with amelogenin signals becoming more intense in all three genotypes. (O–P) Enamelin expression is evident in the wild type, trace amounts of expression in the *Enam* ^+/−^ molars (S–T) but complete absence in *Enam* ^−/−^ molars (W–X).

Despite the cellular abnormalities, amelogenin was secreted but enamelin was absent in all PN5 and PN14 *Enam* ^−/−^ samples (Fig. 5). In normal development, amelogenin and enamelin are deposited in the enamel space. Because of demineralization during sample processing, the enamelin matrix is not preserved. Therefore in this experiment, amelogenin and enamelin were detected only at the DEJ and ameloblast distal membrane. Enamelin expression was detected in both the wild type and the *Enam* ^+/−^ molars (Fig. 5C, G, O, S). Abnormal ameloblast aggregations and cyst-like bulges containing amelogenin were observed in *Enam* ^−/−^ molars (Fig. 5I, J, U, V). Ameloblasts demonstrated premature and excessive apoptotic activities as early as PN5 in *Enam* ^−/−^ molars (Fig. 2K–N). In a previous report, we observed apoptosis in some ameloblasts in postsecretory transition along the mesial and distal cuspal slopes of *Enam* ^+/−^ molars, but the levels were not different from those observed in the wild type molars [16].

### Ultrastructural analyses

**Figure 6:**
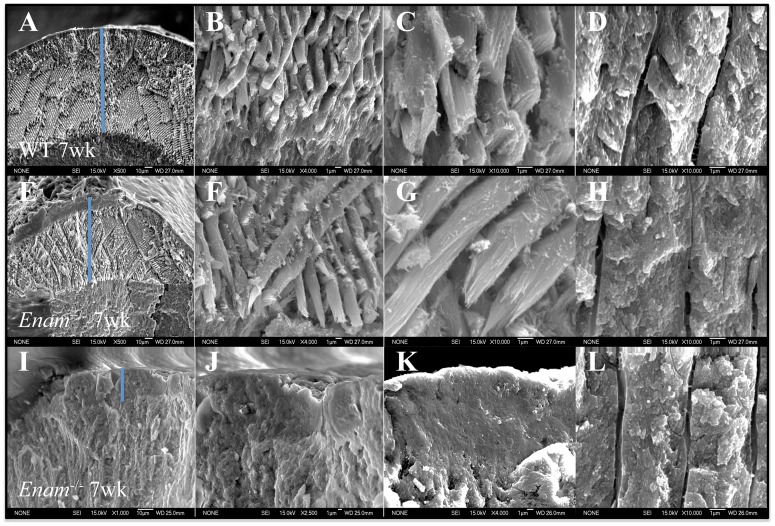
SEM of enamel layer, enamel rods, DEJ, and dentin from 7-week-old mandibular incisors of wild type (A–D), *Enam* ^+/−^ (E–H) and *Enam* ^−/−^ (I–L). Although no major differences in enamel and dentin seem to exist in the light microscope, under the higher resolution of SEM, the mineral crystals in the enamel rods of *Enam* ^+/−^ samples are more distinct and they fracture differently from the wild type crystals (C, G). Amorphous enamel surface (I), unevenly thin enamel layer absent of decussating rods (J, K) but seemingly normal dentin mineralization (L) are observed consistently in *Enam* ^−/−^ incisor samples. Amorphous mineral deposition and occasionally plate-like minerals are seen in the pseudo enamel layer of *Enam* ^−/−^ mouse samples. Blue line indicates enamel thickness.

**Figure 7:**
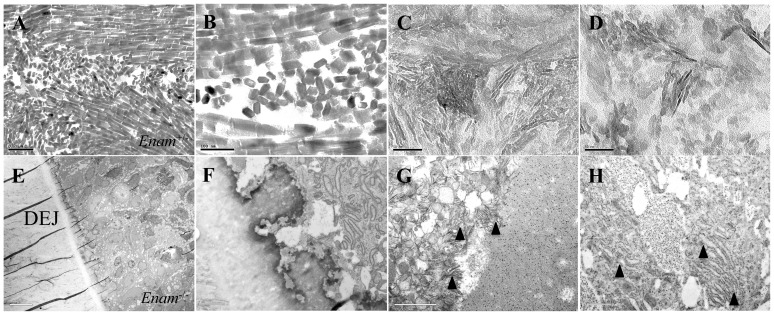
TEM crystal morphology and expression of amelogenin in *Enam* ^+/−^ and *Enam* ^−/−^ mice. (A–D) Longitudinal and cross sections of forming enamel crystals in *Enam* ^+/−^ molars where typical enamel crystals (A–B) and less well-defined, amorphous crystals (C–D) can be detected in different areas of the developing enamel. (E, F) Low magnification of ameloblasts and the dystrophic enamel from PN7 *Enam* ^−/−^ molar showing a scalloped zone of irregular, mineralized tissue at the dentinoenamel junction (DEJ) where layered mineral of different textures is apparent. (G–H) Immunogold labeling for amelogenin appeared irregularly associated with mineralized masses/layers and between disorganized ameloblasts at occasional ectopic calcification sites (G), or as small and large pools of amelogenin between ameloblasts (H). Ectopic, mineralizing cellular debris (arrowheads in G), is also consistently observed. Enlarged rough endoplasmic reticulum is readily observed in the ameloblasts (arrowheads in H). Bars = 100 nm in A–B, 50 nm in C–D, 10 µm in E, and 1 µm in F–H.

A dosage effect of enamelin absence was observed in 7-week-old incisors under SEM (Fig. 6). Organized rods were present in the *Enam* ^+/−^ samples but these rods fractured differently and were spaced farther apart compared to wild type samples (Fig. 6B, C, F–G). There were no organized crystals in the enamel space of *Enam* ^−/−^ incisors and the tooth surfaces were rough and highly irregular (Fig. 6J, K). There were no appreciable differences in dentin structure among all samples (Fig. 6D, H, L). Using TEM, organized crystals of hexagonal shape were present in some areas of the *Enam* ^+/−^ samples (Fig. 7A–B) while in different areas heterogeneous mineral phases were observed in the outermost part of the developing enamel layer (Fig. 7C–D). Amelogenin was produced by *Enam* ^−/−^ ameloblasts but it pooled in the enamel space as well as inside and between ameloblasts. Abnormal mineral mixed with amelogenins were present extracellularly (Fig. 7E–H). Enamel and dentin hardness in *Enam* ^+/−^ mandibular incisors was no different from wild type (data not shown). There was no true enamel in the *Enam* ^−/−^ samples, making hardness impossible to measure.

### *Enam* transgenic models

**Figure 8:**
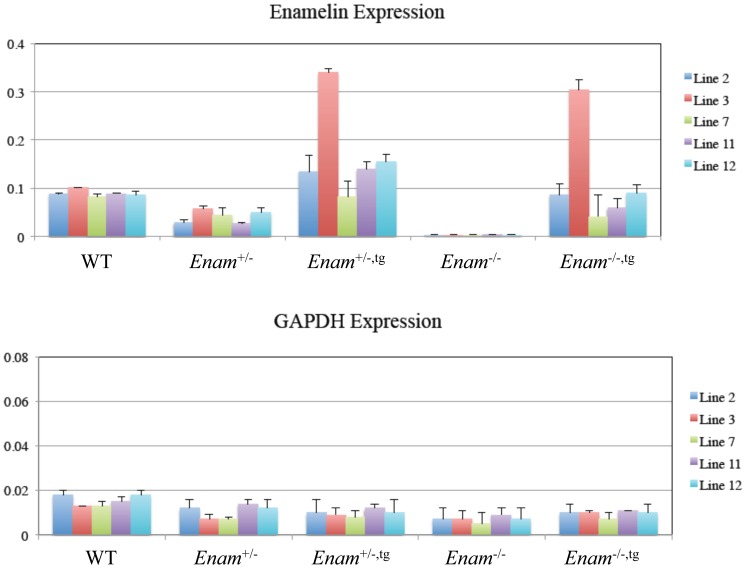
Enamelin transgene expression as determined by ELISA. Graph A presents the enamelin expression as detected by mENAM^223–236^ anti-peptide antibodies in wild type, *Enam* ^+/−^, *Enam* ^−/−^, *Enam* ^+/−,tg^, *Enam* ^−/−,tg^ mouse molars from 5 different transgenic lines. Lines 7 and 11 are low expressers, lines 2 and 12 are medium expressers, and line 3 is high expresser. Graph B demonstrates GAPDH expression levels of test samples, which were assessed to be comparable among all test samples.

The mouse amelogenin (*Amelx*) 5′ and 3′ regulatory regions and the *Enam* coding sequences were separately amplified from genomic DNA template using oligonucleotide primers that introduced rare 8 base restriction sites (Fig. S1). The amplification products were ligated into the pCR2.1TOPO cloning vector and subsequently excised and combined to produce the *Enam* transgenic construct that included 4655 bp of *Amelx* 5′ promoter sequence extending into exon 2 but short of amelogenin translation imitation site, the complete *Enam* cDNA sequence including translation initiation and termination sites, and 1143 bp of 3′ *Amelx* sequence that included three transcription termination signals (Fig. S2). The construct was characterized by DNA sequencing, which verified that the sequence was unaltered by the cloning process (Fig. S3). The transgene construct was excised from the vector by *Not*I and *Srf*I digestion and used for blastocyst microinjection, which yielded 13 positive founder lines. The strategy used for breeding and genotyping the transgenic mice is demonstrated in Fig. S4. Three out of the 13 transgenic lines never produced offspring, 3 female founders were poor breeders and unable to produce sufficient transgene-positive offspring to keep the line viable, one founder line never produced positive pups and was terminated after 6 litters, and one founder line generated pups with almost undetectable transgene levels was also excluded. Multiple viable transgenic lines were evaluated for their levels of enamelin transgene expression in developing teeth using ELISA protein quantification methods. Lines 7 and line 11 expressed the transgenes at low levels, and were designated 7^(L)^ and 11^(L)^. Lines 2 and 12 expressed their transgene at medium levels, and were designated 2^(M)^ and 12^(M)^. These transgenes were still expressed at less than half of normal *Enam* expression levels. Line 3 expressed its transgene at a higher level than normal *Enam* expression and was designated 3^(H)^ (Fig. 8A). GAPDH levels were determined to be similar in all genotypes (Fig. 8B). Expression of *Enam* transgenes at medium or high levels in wild type mice altered both the appearance and surface texture of the enamel (Fig. S5). Transgenic overexpression of *Enam* produced whitish calcospherites on the mandibular incisor surface, which contributed to the chalky white appearance of those teeth (Fig. S5 J, O, T).

### *Enam* transgene rescue

**Figure 9:**
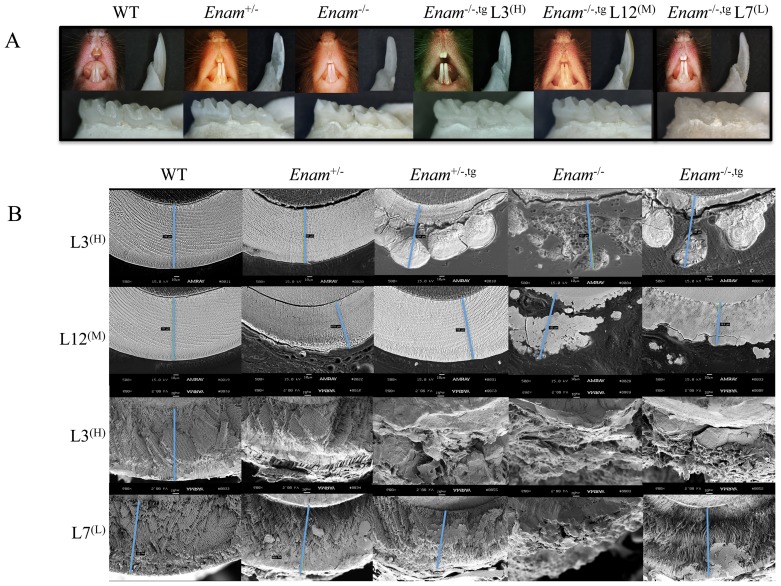
Comparison of incisor enamel in wild type mice with incisor enamel from littermates of a cross between *Enam* ^+/−,tg^ and *Enam* ^−/−^ mice. (A) The *Enam* ^+/−^ mice without the *Enam* transgene have chalky enamel that readily chips from dentin, although it has slightly decreased thickness and seemingly normal rod organization except for the outer enamel layer. The *Enam* ^−/−^ mouse has a mineral crust covering dentin that is very thin, and shows none of the characteristic of enamel. (B, BEI and fractured surface SEM of line 12). The *Enam* ^+/−,tg^ from line12^(M)^ had normal looking enamel that was similar to the wild type in thickness, prism pattern, and resistance to wear but in line 12^(M)^ *Enam* ^−/−,tg^ incisors and molars, the structural defect of enamel was not recovered based on SEM observation. (B, lines 3 and 7) The fractured surface SEMs of lines 3^(H)^ and 7^(L)^ in the lower half of panel B show the thickness of incisor enamel fractured at the alveolar crest. No rescue of enamel defect can be observed in line 3^(H)^ *Enam* ^−/−,tg^ but partial recovery of thickness and structure can be seen in line 7^(L)^ *Enam* ^−/−,tg^. Blue line depicts thickness of the presumed enamel layer.

**Figure 10:**
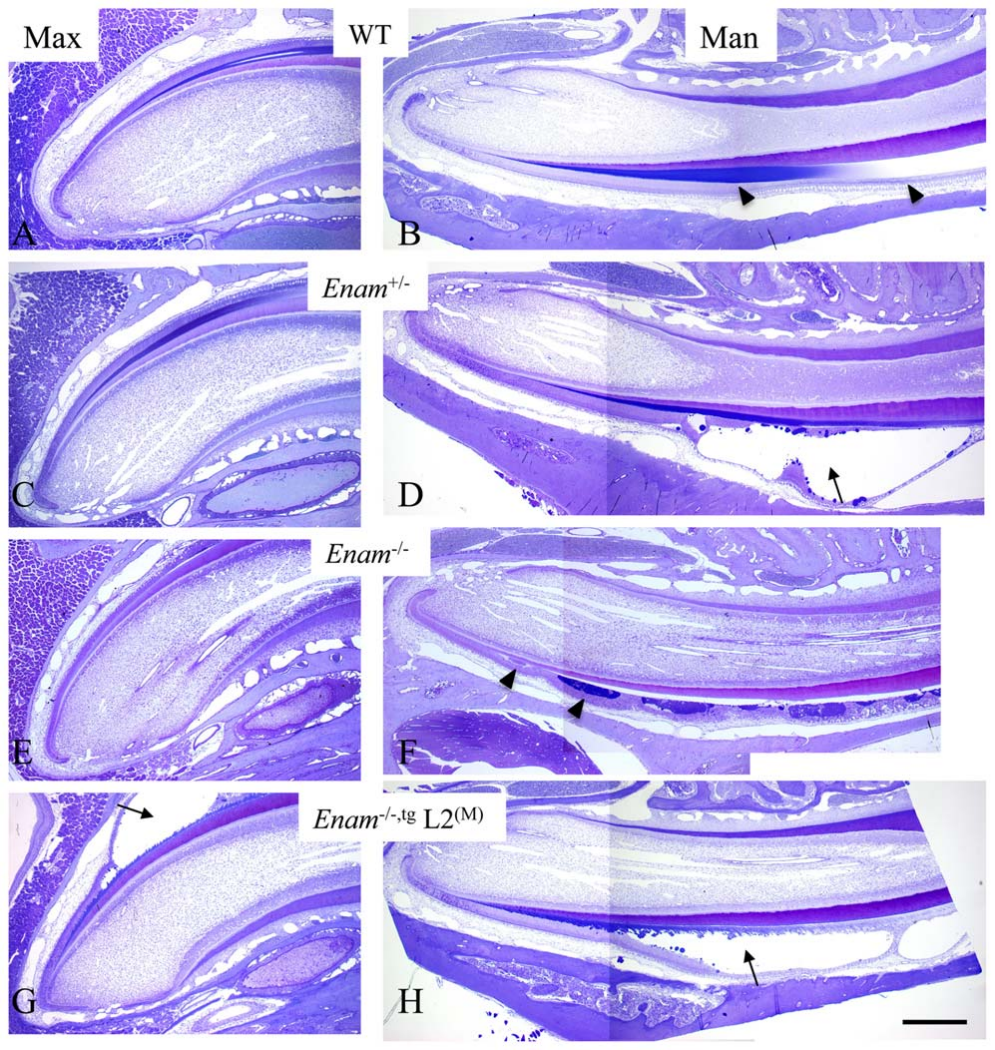
Survey of enamel formation in presecretory, secretory and early maturation stages on maxillary and mandibular incisors from 7-week-old mice. (A, B) Wild type incisors showed normal matrix deposition and enamel development. Matrix protein stained blue in the enamel space during secretory stage was absorbed in the maturation stage allowing crystal maturation to take place (arrowheads). (C) Maxillary incisors in *Enam^+/−^* mice appear similar to wild type. (D) However, the mandibular incisors in these mice consistently demonstrated disturbed ameloblasts resulting in cyst formation within the enamel organ beginning in late secretory stage (arrow). (E) In *Enam^−/−^* maxillary incisors, there was no apparent enamel matrix deposition; (F) in the mandibular incisors, soon after the onset of the secretory stage, ameloblasts showed pooling of secreted material at their apices and gradual loss of polarity and organization within the enamel organ (arrowheads). (G, H) In line 2^(M)^, *Enam^−/−^* ^,tg^ incisors, ameloblasts were polarized and deposited matrix during early secretory stage. Soon after that ameloblast layer became detached and cystic formation was evident on both maxillary and mandibular incisors (arrows). These 0.5 µm thick toluidine blue stained sections of EDTA-decalcified incisors were obtained from mice perfused with 2.5% GA and embedded in Epon. Bar = 500 µm for all panels.

Five founder lines selected by their demonstration of germline transmission and *Enam* ^+/+,tg^ expression levels were crossed with *Enam* ^−/−^ mice to generate offspring with 4 different genotypes, *Enam* ^+/−^, *Enam* ^−/−^, *Enam* ^+/−,tg^, *Enam* ^−/−,tg^ (Fig. S4). Developing teeth from offspring with the correct genotype were then compared. Blunting of the incisal edge and cuspal tips of molars suggested altered enamel hardness in *Enam* ^+/−^, *Enam* ^−/−^, and *Enam* ^−/−,tg^ mice (Fig. 9A). Comparing offspring from line 12^(M)^, line 7^(L)^ and the wild type, the enamel phenotype was partially recovered when enamelin transgene was expressed in the *Enam* ^−/−^ background [Fig. 9B, lines 12^(M)^ and 7^(L)^]. When higher than normal levels of enamelin were expressed in the *Enam* ^−/−^ background, such as in the case of line 3^(H)^, the enamel covering the molars and incisors appeared to be abnormal in thickness and lacked prism structure as revealed by backscatter and conventional SEM analyses (Fig. 9B, line 3). Close to normal enamel density, structure and thickness were observed only in *Enam^+/−^* ^,tg^ of line 12^(M)^ while in the same genetic background *Enam^+/−^* ^,tg^ of line 7^(L)^ the enamel prism and thickness was only partially restored (Fig. 9B). Also in line 7^(L)^, recovery was best near the DEJ where enamel crystals seemed to form normally followed by the usual decussated rods with surrounding interrod material although outer enamel was never rescued. Histologically, the most apparent finding was ameloblast pathology and cyst formation in the absence of enamelin, which was evident in both the maxillary and mandibular incisors of the *Enam* ^−/−^ and *Enam* ^−/−,tg^ mice but only the mandibular incisors of the *Enam* ^+/−^ mice (Fig. 10). Introducing enamelin at a low expression level did not alleviate the pathologic changes of ameloblasts (Fig. 10G–H).

**Figure 11:**
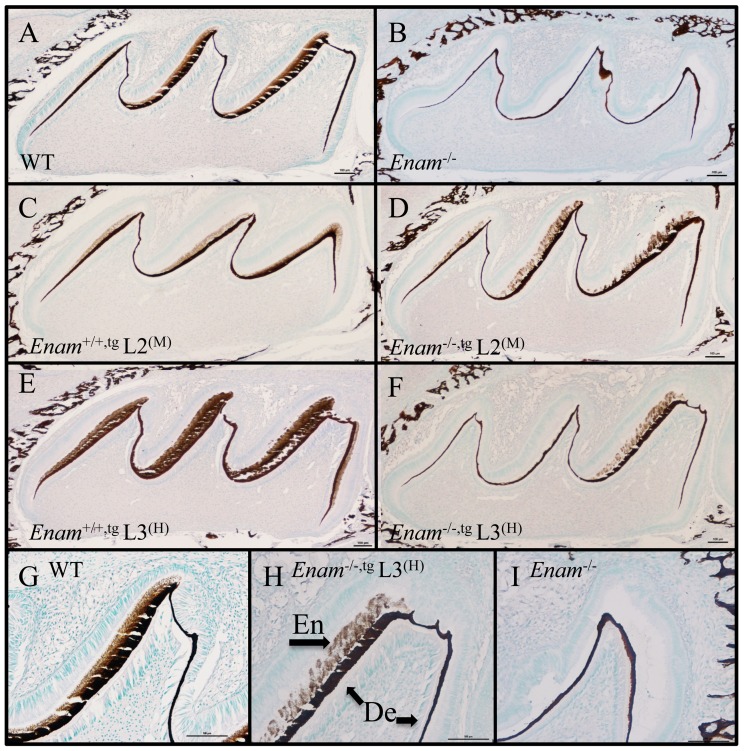
Von Kossa staining of PN4 first molars from wild type, *Enam* ^−/−^, *Enam* ^+/−,tg^, and *Enam* ^−/−,tg^. (A) Positive von Kossa stain was present in bone, dentin and enamel of wild type molars. (B) Although dentin was positively stained, there is no enamel layer evident in *Enam* ^−/−^ molars. Molars of *Enam* ^+/−,tg^ mice from lines 2^(M)^ (C) and 3^(H)^ (E) both demonstrated positively stained enamel and dentin layers. Molars of *Enam* ^−/−,tg^ mice from line 2^(M)^ (D) showed positively stained enamel in all cusps but mice from line 3^(H)^ (F) showed positively stained enamel only on major cusps. A positively stained enamel layer is evident on the major cusp slope of molars from wild type (G) and *Enam* ^−/−,tg^ mice in line 3^(H)^ (H) but not in *Enam* ^−/−^ mice (I). En: enamel, De: dentin. Bar = 100 µm.

Positive von Kossa reaction of the enamel and dentin of the wild type molars were evident (Fig. 11A, G), while only the dentin layer demonstrated positive reaction in the *Enam* ^−/−^ molars (Fig. 11B, I). Molars from PN4 line 2^(M)^ and line 3^(H)^ *Enam^+/+^* ^,tg^ *and Enam* ^−/−,tg^ were also tested. Although enamelin transgene expression in line 2^(M)^ and line 3^(H)^ did not rescue the enamel thickness or structures, von Kossa staining detected the presence of mineral in the presumed enamel space of the developing molars (Fig. 11C, E). Positive reaction in the enamel layer of the *Enam^+/+^* ^,tg^ molars from line 2^(M)^ (Fig. 11C) and line 3^(H)^ (Fig. 11E) revealed appreciable differences in staining intensity. Enamel mineralization as demonstrated by von Kossa positive reaction was present in *Enam* ^−/−,tg^ samples from line 2^(M)^ (Fig. 11D, H) and line 3^(H)^ (Fig. 11F, H).

## Discussion

*Amelogenesis imperfecta* is a diverse group of disorders affecting dental enamel formation; when clinical phenotypes and mode of inheritance are considered, 15 subtypes can be distinguished [19]. Single allele mutations of *ENAM* result in hypoplastic enamel where the affected enamel is thin and rough and the teeth are yellow and sensitive to thermal changes. Mutations in both alleles often lead to complete absence of enamel. Patients with such a condition require extensive and frequent dental care, which causes a significant psychological and financial burden. To improve diagnosis, treatment and long-term prognosis of patients with AI, understanding the genetic control of amelogenesis is paramount. The enamelin knockout and transgenic mouse models described here make possible the study of human hypoplastic AI arising from enamelin gene defects.

In this study, we conclude that enamelin is essential for the formation of dental enamel with its distinctive crystal and the prism structures. Characterization of the enamelin gene-targeted mouse models reveals the essential function of enamelin in maintaining ameloblast integrity and directing the assembly of enamel ultrastructure. Based upon the analysis of the knockout mouse model, we determined the temporal and spatial expression of enamelin, characterized the structural and cellular defect of amelogenesis, which provided the pathologic basis of enamel hypoplasia. Furthermore, the study of selected enamelin transgenic mouse lines, determination of transgene expression by protein quantification methods, and introduction of enamelin transgenes into the knockout background to recover enamel defects demonstrated a dosage effect of enamelin.

Considering that regulatory elements may reside in intronic regions, introns 1 and 2 were preserved in the design of *Enam* KO/*lacZ* KI construct. Enamelin expression as demonstrated by β-galactosidase staining is specific to the developing tooth organ, restricted to the secretory and early maturation stage ameloblasts. Expression of enamelin in odontoblasts or osteoblasts was not observed. In the developing molars from the *Enam* ^−/−^ mouse, the ameloblast layer becomes increasingly crowded as tooth formation progresses. By PN5 small groups of ameloblasts lose contact with the matrix covering dentin and form nodules within the stratum intermedium. Ameloblasts continue to synthesize and secrete amelogenin, but show atypical features including expanded endoplasmic reticulum. An extracellular layer of organic material abundant in amelogenins is deposited on the surface of normal appearing dentin although no mineralization takes place. Mineral formation at the ameloblast-matrix interface (mineralization front) is absent and no enamel crystal ribbons can be observed at the early secretory stage (PN5). At the secretory stage, the dentinoenamel junction (DEJ) appears to be highly irregular as shown in Fig. 7F. Amorphous, granular deposits of pathological calcification are observed atop the DEJ and between ameloblasts. In the *Enam* ^+/−^ mice, the enamel crystals have the classic hexagonal appearance but are less densely packed than in wild type. Ameloblasts in the *Enam* ^−/−^ mice have poorly formed Tomes' processes and undergo pathological alterations that lead to ectopic calcifications and the formation of a thin, highly irregular mineral crust instead of true enamel.

There exist appreciable differences among the defective enamel found in amelogenin, ameloblastin and enamelin knockout mice. Although the absence of amelogenin [20] and ameloblastin [4] also lead to hypoplastic enamel defects, the degree of hypoplasia is most severe in enamelin knockout mice, which produced no true enamel. In case of amelogenin absence, ameloblasts lacking Tomes' processes are able to make a thin layer of enamel that is 10–20% the thickness typical of wild type enamel. When mutant ameloblastin *Ambn* **^Δ^** ^5–6^ is present, the ameloblasts detach from the underlying matrix at the start of the secretory stage and generate a thin enamel with irregular prism pattern [21], [22]. When enamelin is absent, ameloblasts soon become pathological, unable to adhere to underlining surface, and they prematurely undergo apoptosis [16]. Cystic changes often occur at what is normally the start of the secretory stage, which was most apparent on the mesial cuspal slope of the first molar and the enamel organ of the mandibular incisor of the PN7 *Enam* ^−/−^ mice. In the *Enam* ^−/−^ mouse incisors, the matrix-like materials pooled irregularly and primarily within the enamel organ resulting in a very thin enamel space. In the *Enam* ^+/−^ mouse mandibular incisors, the cysts appear late in the secretory stage and carry forward into the maturation stage, which causes additional problems to getting full maturation of the enamel. It ends up chalky white and soft on the erupted portion of the tooth. These observations suggest that cell-matrix interactions during enamel formation is a plausible regulatory mechanism of ameloblast activities and the major enamel matrix proteins, amelogenin, ameloblastin and enamelin are likely conducting different functions in a synchronized fashion to produce well organized and fully mineralized dental enamel.

Introducing appropriate transgenes capable of expressing proteins at the quantity comparable to endogenous amounts in a null background to rescue dental defects has been demonstrated by Kulkarni et al., Chun et al., and Gibson et al. In the case of DSPP absence, re-introducing DSP only improves the dentin volume but not the mineral density [23]. In the case of *Ambn* **^Δ^** ^5–6^ partial deletion mutant, expression of its transgene at somewhat higher than the normal levels almost fully rescues the enamel defects in the mutant background [24]. Most interestingly, mouse amelogenin180-87 cleavage products rescues enamel mineral density and increases thickness while introducing both amelogenin180-87 and leucine-rich amelogenin peptide, which contains residue 1–33 plus 13 residues from the most C-terminus of the amelogenin180, restores enamel prism structure and further improves enamel thickness in the *Amelx* knockout mice. This study demonstrated functional importance of different amelogenin cleavage products in enamel formation [20]. In our study, complete rescue of the enamel defect in the enamelin null background was not achieved by introducing three different levels of transgene expression from the amelogenin promoter whereas typical enamel thickness and prism structures were observed when near normal levels of the transgene were introduced into the heterozygous *Enam* ^+/−^ background where partial defects were present. The collective results suggest there exists a very tight control of enamelin dosage in order to achieve proper enamel formation. This is further demonstrated by the mineralization defects seen in wild type mice starting with the lowest expression levels of “extra” enamelin (Fig. S5).

The most intriguing finding of this study is that in the *Enam* ^−/−^ mice, there is absence of proper enamel matrix accumulation and organized enamel crystals growth resulting in no true enamel formation. The mineralized crust later formed on the dentin surface of the *Enam* ^−/−^ mice is likely a product of pathologic calcification. Re-introduction of enamelin allowed initial mineralization to take place at the DEJ during early secretory stage, although the specific dosage of the introduced enamelin dictates how well the enamel ultrastructure is recovered. Such evidence and the observation of restricted localization pattern of C-terminus enamelin have led us to hypothesize that enamelin is a critical matrix molecule in promoting crystal initiation and elongation during a specific stage of amelogenesis. Furthermore, ameloblast integrity is jeopardized in the absence of enamelin, which suggests the potential involvement of enamelin in maintaining a functional cell-to-matrix interface. Thus, in the absence of enamelin, ameloblast Tomes' process, mineralization front and true enamel formation cannot be attained. Dental enamel forms by the deposition of characteristic thin, non-crystalline, mineral ribbons along a mineralization front that are physically closely associated with the secretory surfaces of the ameloblast plasma membrane. The mineralization front apparatus is the key to enamel formation [25]. Following the formation of initial enamel, enamel matrix components such as amelogenin, enamelin and ameloblastin may self-assemble into nanostructures allowing crystal elongation and organization to take place repetitively with precision to form rods and interrod enamel which subsequently arranged into an intricate decussating pattern [26]. To advance our understanding of the basic mechanism of biomineralization, it will be important to visualize the mineralization front, to discover the pattern of the matrix protein assembly, and to understand their functional relationship with the calcium phosphate phases deposited within the extracellular enamel space.

## Supporting Information

Figure S1 **Amplification and cloning of** ***Enam*** **transgene (Tg) components.** ***Top:*** Sequences of the six PCR primers used to amplify target sequences and to introduce rare (8 base cutter) restriction sites. ***Middle:*** The *AmelX* promoter (5′*AmelX*, 4655 bp), the *Enam* cDNA (*Enam*, 3845 bp), and *AmelX* downstream (3′*AmelX*, 1143 bp) sequence. ***Bottom:*** the three amplification products were ligated into pCR2.1-TOPO (3931 bp). Recombinant plasmids having the 5′ ends of the PCR products on the *NotI* side of the vector were used to construct the *Enam* transgene.
(DOCX)

Figure S2 **Constructing the** ***Enam*** **transgene.** The *Enam* transgene expresses from the *AmelX* promoter. Transcription initiates in the 5′*AmelX* region at the start of the exon 1, which is non-coding. Intron 1 (1277 bp) of *AmelX* is removed by RNA splicing. The *AscI* site connects 5′*AmelX*, including 10 nucleotides in exon 2, to the *Enam* cDNA sequence (3845 bp). The splice junction at the start of exon 2 is indicated by hash marks in the expanded sequence surrounding the *AscI* site. The *Enam* cDNA sequence is immediately downstream of the *AscI* site and is in lower case and boxed. The *AmelX* and *Enam* translation initiation codons are underlined.
(DOCX)

Figure S3 **Mouse** ***Enam*** **transgene construct starting with pCR2.1-TOPO vector.** Restriction sites used during construction are in bold and underlined. The *Enam* translation initiation and termination codons are in bold. 1–3899 is vector sequence ending at the *NotI* (GCGGCCGC) site; 3900–8538 is from the *AmelX* 5′ transcription regulatory (promoter) region (4639 bp) ending at the introduced *AscI* (GGCGCGCC) site; 8547–12391 is *Enam* cDNA sequence (3845 bp) ending at the introduced *SgfI* (GCGATCGC) site; 12400–13527 is from the *AmelX* 3′ region (1127 bp), which contains multiple transcription termination signals and ends at the introduced *SrfI* (GCCCGGGC) site.
(DOCX)

Figure S4 **Breeding and genotyping strategy.** ***A:*** Breeding Strategy. *Enam* ^+/+,tg^ (A) offspring were mated with an *Enam* ^−/−^ mouse (B). The F1 offspring (C & D) were genotyped by tail biopsy. F1 mice positive for the transgene (D) are mated to *Enam* ^−/−^ mice producing an F2 generation with four genotypes (B, C, D, E), which are identified by genotyping. Such breeding allowed us to use littermates to compare the phenotypes of four different genotypes (all except the wild-type). ***B:*** Genotyping primers. Two PCR primer pairs (1 and 2; 3 and 4) were used to identify mice carrying an *Enam* transgene (Tg). A primer pair (5 and 6) specifically detected the NSL β–gal in mice carrying the *Enam* knockout construct. ***C:*** Agarose gels showing the different patterns of PCR amplification products that determined the genotype of each offspring. Please note that “4&5” represents enamelin exon 4 and exon 5 coding region, which was amplified using Enam 4&5F and Enam 4&5R primers.
(DOCX)

Figure S5 **Incisor and molar teeth of wild type and enamelin transgenic mice from lines 12, 2, and 3.** (A–E) Representative photographs of 7 weeks old wild type mouse, (F–J) transgenic medium expressers line 12^(M)^, (K–O) line 2^(M)^, and high expresser (P–T) line 3^(H)^ mice. These illustrations include (A, F, K, P) intraoral photograph, (B, G, L, Q) distal view of the right mandibular incisor, (C, H, M, R) mesial view of right mandible, and (D, I, N, S) mesial view of right molars. Series of labial views of the mandibular incisor from mice shown in (E) wild type, (J) line 12^(M)^, (O) line 2^(M)^; and (T) line 3^(H)^. The magnification is 4× in (A, F, K, P); 6× in (B, G, L, Q); 3× in (C, H, M, R); 6× in (D, I, N, S). The magnifications of labial views from left to right in each series of E, J, O and T are 6×, 9×, 12×, 18× and 24×.
(DOCX)
