# Supplementary figures and images for: Enamelin Is Critical for Ameloblast Integrity and Enamel Ultrastructure Formation
Source: PLoS One. 2014 Mar 6;9(3):e89303. doi: 10.1371/journal.pone.0089303 (PMC3945975; doi:10.1371/journal.pone.0089303)

| 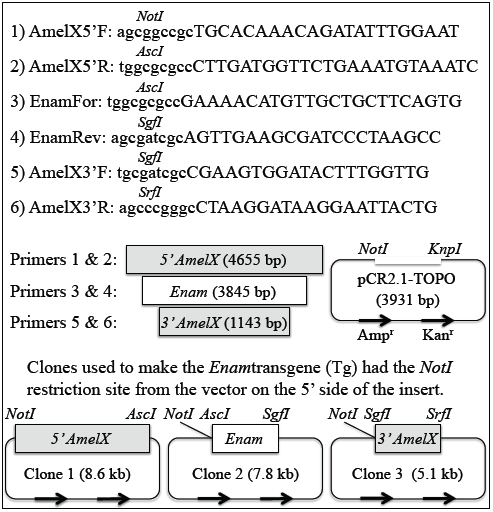 |
| --- |

Supplement: Figure S1 — Amplification and cloning of Enam transgene (Tg) components. Top: Sequences of the six PCR primers used to amplify target sequences and to introduce rare (8 base cutter) restriction sites. Middle: The AmelX promoter (5′AmelX, 4655 bp), the Enam cDNA (Enam, 3845 bp), and AmelX downstream (3′AmelX, 1143 bp) sequence. Bottom: the three amplification products were ligated into pCR2.1-TOPO (3931 bp). Recombinant plasmids having the 5′ ends of the PCR products on the NotI side of the vector were used to construct the Enam transgene. (DOCX) [file pone.0089303.s001.docx]

| 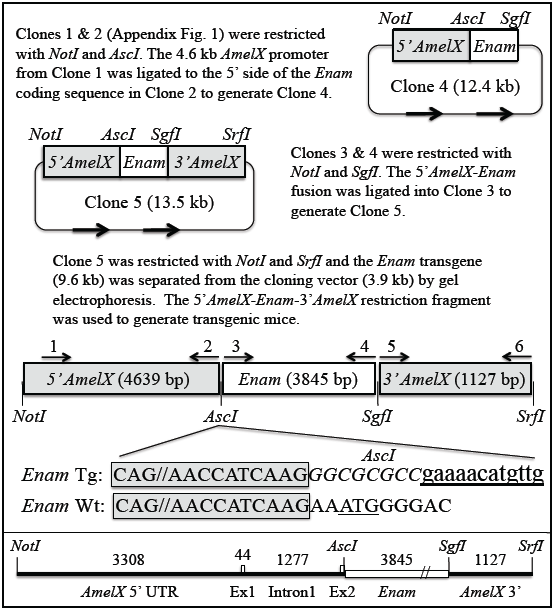 |
| --- |

Supplement: Figure S2 — Constructing the Enam transgene. The Enam transgene expresses from the AmelX promoter. Transcription initiates in the 5′AmelX region at the start of the exon 1, which is non-coding. Intron 1 (1277 bp) of AmelX is removed by RNA splicing. The AscI site connects 5′AmelX, including 10 nucleotides in exon 2, to the Enam cDNA sequence (3845 bp). The splice junction at the start of exon 2 is indicated by hash marks in the expanded sequence surrounding the AscI site. The Enam cDNA sequence is immediately downstream of the AscI site and is in lower case and boxed. The AmelX and Enam translation initiation codons are underlined. (DOCX) [file pone.0089303.s002.docx]

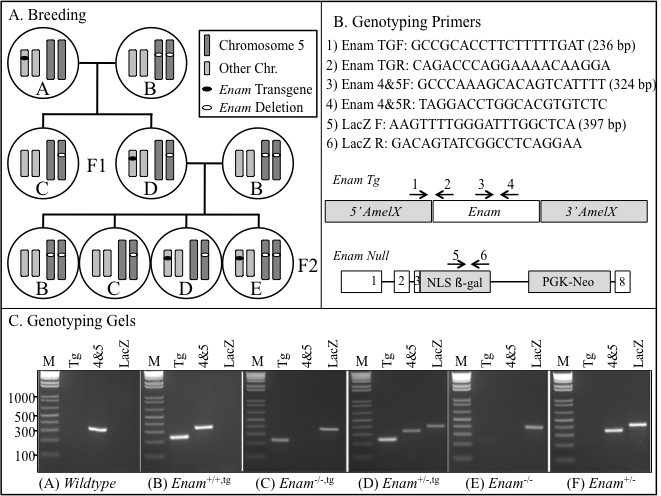


|  |
| --- |

Supplement: Figure S4 — Breeding and genotyping strategy. A: Breeding Strategy. Enam +/+,tg (A) offspring were mated with an Enam −/− mouse (B). The F1 offspring (C & D) were genotyped by tail biopsy. F1 mice positive for the transgene (D) are mated to Enam −/− mice producing an F2 generation with four genotypes (B, C, D, E), which are identified by genotyping. Such breeding allowed us to use littermates to compare the phenotypes of four different genotypes (all except the wild-type). B: Genotyping primers. Two PCR primer pairs (1 and 2; 3 and 4) were used to identify mice carrying an Enam transgene (Tg). A primer pair (5 and 6) specifically detected the NSL β–gal in mice carrying the Enam knockout construct. C: Agarose gels showing the different patterns of PCR amplification products that determined the genotype of each offspring. Please note that “4&5” represents enamelin exon 4 and exon 5 coding region, which was amplified using Enam 4&5F and Enam 4&5R primers. (DOCX) [file pone.0089303.s004.docx]

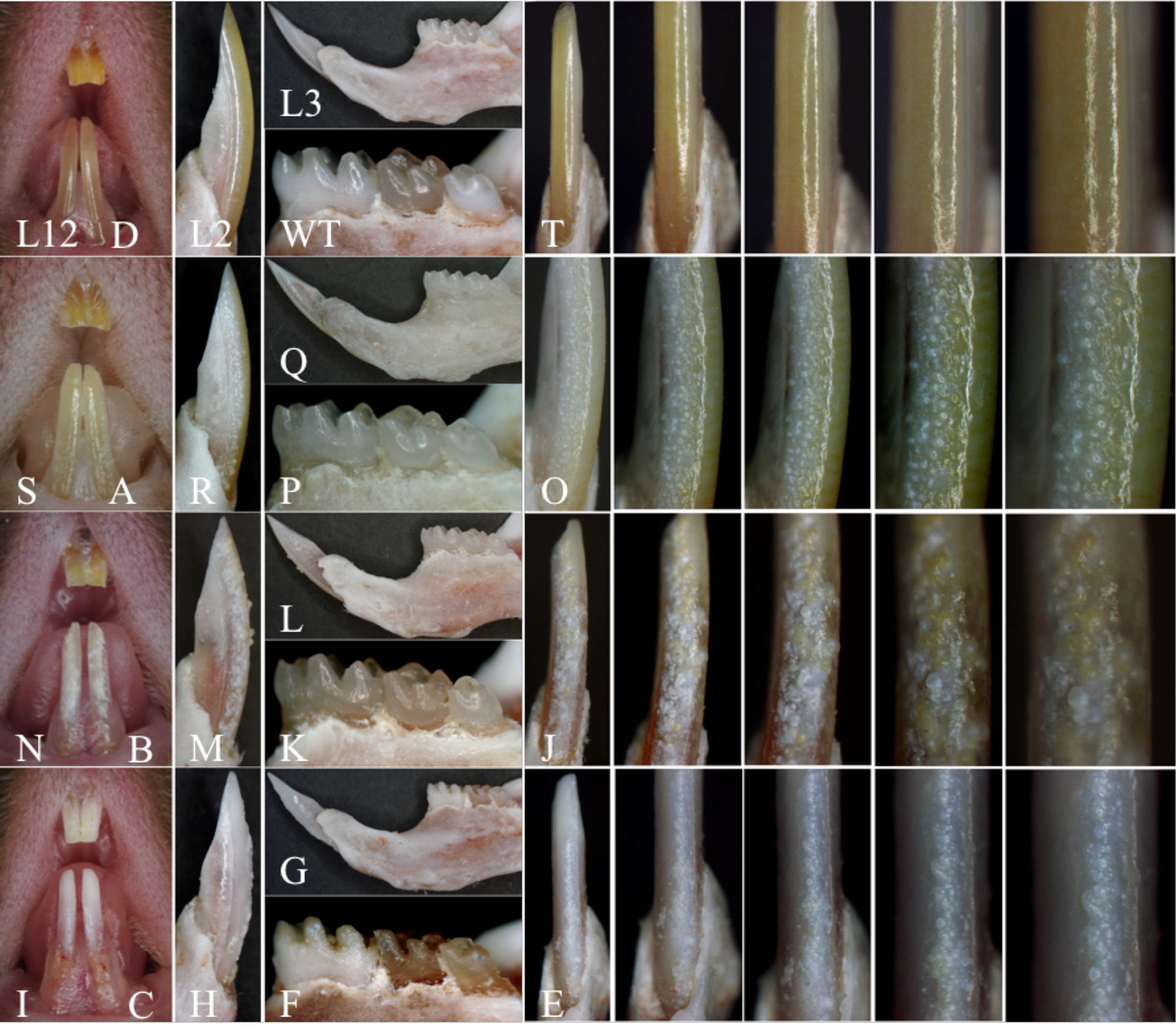

Supplement: Figure S5 — Incisor and molar teeth of wild type and enamelin transgenic mice from lines 12, 2, and 3. (A–E) Representative photographs of 7 weeks old wild type mouse, (F–J) transgenic medium expressers line 12(M), (K–O) line 2(M), and high expresser (P–T) line 3(H) mice. These illustrations include (A, F, K, P) intraoral photograph, (B, G, L, Q) distal view of the right mandibular incisor, (C, H, M, R) mesial view of right mandible, and (D, I, N, S) mesial view of right molars. Series of labial views of the mandibular incisor from mice shown in (E) wild type, (J) line 12(M), (O) line 2(M); and (T) line 3(H). The magnification is 4× in (A, F, K, P); 6× in (B, G, L, Q); 3× in (C, H, M, R); 6× in (D, I, N, S). The magnifications of labial views from left to right in each series of E, J, O and T are 6×, 9×, 12×, 18× and 24×. (DOCX) [file pone.0089303.s005.docx]
