# Supplementary material for: Enamelin Is Critical for Ameloblast Integrity and Enamel Ultrastructure Formation
Source: PLoS One. 2014 Mar 6;9(3):e89303. doi: 10.1371/journal.pone.0089303 (PMC3945975; doi:10.1371/journal.pone.0089303)
Supplement: Figure S3 — Mouse Enam transgene construct starting with pCR2.1-TOPO vector. Restriction sites used during construction are in bold and underlined. The Enam translation initiation and termination codons are in bold. 1–3899 is vector sequence ending at the NotI (GCGGCCGC) site; 3900–8538 is from the AmelX 5′ transcription regulatory (promoter) region (4639 bp) ending at the introduced AscI (GGCGCGCC) site; 8547–12391 is Enam cDNA sequence (3845 bp) ending at the introduced SgfI (GCGATCGC) site; 12400–13527 is from the AmelX 3′ region (1127 bp), which contains multiple transcription termination signals and ends at the introduced SrfI (GCCCGGGC) site. (DOCX) [file pone.0089303.s003.docx]

1 AAGGGCGAAT TCCAGCACAC TGGCGGCCGT TACTAGTGGA TCCGAGCTCG GTACCAAGCT

61 TGGCGTAATC ATGGTCATAG CTGTTTCCTG TGTGAAATTG TTATCCGCTC ACAATTCCAC

121 ACAACATACG AGCCGGAAGC ATAAAGTGTA AAGCCTGGGG TGCCTAATGA GTGAGCTAAC

181 TCACATTAAT TGCGTTGCGC TCACTGCCCG CTTTCCAGTC GGGAAACCTG TCGTGCCAGC

241 TGCATTAATG AATCGGCCAA CGCGCGGGGA GAGGCGGTTT GCGTATTGGG CGCTCTTCCG

301 CTTCCTCGCT CACTGACTCG CTGCGCTCGG TCGTTCGGCT GCGGCGAGCG GTATCAGCTC

361 ACTCAAAGGC GGTAATACGG TTATCCACAG AATCAGGGGA TAACGCAGGA AAGAACATGT

421 GAGCAAAAGG CCAGCAAAAG GCCAGGAACC GTAAAAAGGC CGCGTTGCTG GCGTTTTTCC

481 ATAGGCTCCG CCCCCCTGAC GAGCATCACA AAAATCGACG CTCAAGTCAG AGGTGGCGAA

541 ACCCGACAGG ACTATAAAGA TACCAGGCGT TTCCCCCTGG AAGCTCCCTC GTGCGCTCTC

601 CTGTTCCGAC CCTGCCGCTT ACCGGATACC TGTCCGCCTT TCTCCCTTCG GGAAGCGTGG

661 CGCTTTCTCA TAGCTCACGC TGTAGGTATC TCAGTTCGGT GTAGGTCGTT CGCTCCAAGC

721 TGGGCTGTGT GCACGAACCC CCCGTTCAGC CCGACCGCTG CGCCTTATCC GGTAACTATC

781 GTCTTGAGTC CAACCCGGTA AGACACGACT TATCGCCACT GGCAGCAGCC ACTGGTAACA

841 GGATTAGCAG AGCGAGGTAT GTAGGCGGTG CTACAGAGTT CTTGAAGTGG TGGCCTAACT

901 ACGGCTACAC TAGAAGAACA GTATTTGGTA TCTGCGCTCT GCTGAAGCCA GTTACCTTCG

961 GAAAAAGAGT TGGTAGCTCT TGATCCGGCA AACAAACCAC CGCTGGTAGC GGTGGTTTTT

1021 TTGTTTGCAA GCAGCAGATT ACGCGCAGAA AAAAAGGATC TCAAGAAGAT CCTTTGATCT

1081 TTTCTACGGG GTCTGACGCT CAGTGGAACG AAAACTCACG TTAAGGGATT TTGGTCATGA

1141 GATTATCAAA AAGGATCTTC ACCTAGATCC TTTTAAATTA AAAATGAAGT TTTAAATCAA

1201 TCTAAAGTAT ATATGAGTAA ACTTGGTCTG ACAGTTACCA ATGCTTAATC AGTGAGGCAC

1261 CTATCTCAGC GATCTGTCTA TTTCGTTCAT CCATAGTTGC CTGACTCCCC GTCGTGTAGA

1321 TAACTACGAT ACGGGAGGGC TTACCATCTG GCCCCAGTGC TGCAATGATA CCGCGAGACC

1381 CACGCTCACC GGCTCCAGAT TTATCAGCAA TAAACCAGCC AGCCGGAAGG GCCGAGCGCA

1441 GAAGTGGTCC TGCAACTTTA TCCGCCTCCA TCCAGTCTAT TAATTGTTGC CGGGAAGCTA

1501 GAGTAAGTAG TTCGCCAGTT AATAGTTTGC GCAACGTTGT TGCCATTGCT ACAGGCATCG

1561 TGGTGTCACG CTCGTCGTTT GGTATGGCTT CATTCAGCTC CGGTTCCCAA CGATCAAGGC

1621 GAGTTACATG ATCCCCCATG TTGTGCAAAA AAGCGGTTAG CTCCTTCGGT CCTCCGATCG

1681 TTGTCAGAAG TAAGTTGGCC GCAGTGTTAT CACTCATGGT TATGGCAGCA CTGCATAATT

1741 CTCTTACTGT CATGCCATCC GTAAGATGCT TTTCTGTGAC TGGTGAGTAC TCAACCAAGT

1801 CATTCTGAGA ATAGTGTATG CGGCGACCGA GTTGCTCTTG CCCGGCGTCA ATACGGGATA

1861 ATACCGCGCC ACATAGCAGA ACTTTAAAAG TGCTCATCAT TGGAAAACGT TCTTCGGGGC

1921 GAAAACTCTC AAGGATCTTA CCGCTGTTGA GATCCAGTTC GATGTAACCC ACTCGTGCAC

1981 CCAACTGATC TTCAGCATCT TTTACTTTCA CCAGCGTTTC TGGGTGAGCA AAAACAGGAA

2041 GGCAAAATGC CGCAAAAAAG GGAATAAGGG CGACACGGAA ATGTTGAATA CTCATACTCT

2101 TCCTTTTTCA ATTCAGAAGA ACTCGTCAAG AAGGCGATAG AAGGCGATGC GCTGCGAATC

2161 GGGAGCGGCG ATACCGTAAA GCACGAGGAA GCGGTCAGCC CATTCGCCGC CAAGCTCTTC

2221 AGCAATATCA CGGGTAGCCA ACGCTATGTC CTGATAGCGG TCCGCCACAC CCAGCCGGCC

2281 ACAGTCGATG AATCCAGAAA AGCGGCCATT TTCCACCATG ATATTCGGCA AGCAGGCATC

2341 GCCATGGGTC ACGACGAGAT CCTCGCCGTC GGGCATGCGC GCCTTGAGCC TGGCGAACAG

2401 TTCGGCTGGC GCGAGCCCCT GATGCTCTTC GTCCAGATCA TCCTGATCGA CAAGACCGGC

2461 TTCCATCCGA GTACGTGCTC GCTCGATGCG ATGTTTCGCT TGGTGGTCGA ATGGGCAGGT

2521 AGCCGGATCA AGCGTATGCA GCCGCCGCAT TGCATCAGCC ATGATGGATA CTTTCTCGGC

2581 AGGAGCAAGG TGGGATGACA GGAGATCCTG CCCCGGCACT TCGCCCAATA GCAGCCAGTC

2641 CCTTCCCGCT TCAGTGACAA CGTCGAGCAC AGCTGCGCAA GGAACGCCCG TCGTGGCCAG

2701 CCACGATAGC CGCGCTGCCT CGTCCTGCAG TTCATTCAGG GCACCGGACA GGTCGGTCTT

2761 GACAAAAAGA ACCGGGCGCC CCTGCGCTGA CAGCCGGAAC ACGGCGGCAT CAGAGCAGCC

2821 GATTGTCTGT TGTGCCCAGT CATAGCCGAA TAGCCTCTCC ACCCAAGCGG CCGGAGAACC

2881 TGCGTGCAAT CCATCTTGTT CAATCATGCG AAACGATCCT CATCCTGTCT CTTGATCAGA

2941 TCTTGATCCC CTGCGCCATC AGATCCTTGG CGGCAAGAAA GCCATCCAGT TTACTTTGCA

3001 GGGCTTCCCA ACCTTACCAG AGGGCGCCCC AGCTGGCAAT TCCGGTTCGC TTGCTGTCCA

3061 TAAAACCGCC CAGTCTAGCT ATCGCCATGT AAGCCCACTG CAAGCTACCT GCTTTCTCTT

3121 TGCGCTTGCG TTTTCCCTTG TCCAGATAGC CCAGTAGCTG ACATTCATCC GGGGTCAGCA

3181 CCGTTTCTGC GGACTGGCTT TCTACGTGTT CCGCTTCCTT TAGCAGCCCT TGCGCCCTGA

3241 ATTTTGTTAA AATTCGCGTT AAATTTTTGT TAAATCAGCT CATTTTTTAA CCAATAGGCC

3301 GAAATCGGCA AAATCCCTTA TAAATCAAAA GAATAGACCG AGATAGGGTT GAGTGTTGTT

3361 CCAGTTTGGA ACAAGAGTCC ACTATTAAAG AACGTGGACT CCAACGTCAA AGGGCGAAAA

3421 ACCGTCTATC AGGGCGATGG CCCACTACGT GAACCATCAC CCTAATCAAG TTTTTTGGGG

3481 TCGAGGTGCC GTAAAGCACT AAATCGGAAC CCTAAAGGGA GCCCCCGATT TAGAGCTTGA

3541 CGGGGAAAGC CGGCGAACGT GGCGAGAAAG GAAGGGAAGA AAGCGAAAGG AGCGGGCGCT

3601 AGGGCGCTGG CAAGTGTAGC GGTCACGCTG CGCGTAACCA CCACACCCGC CGCGCTTAAT

3661 GCGCCGCTAC AGGGCGCGTC CATTCGCCAT TCAGGCTGCG CAACTGTTGG GAAGGGCGAT

3721 CGGTGCGGGC CTCTTCGCTA TTACGCCAGC TGGCGAAAGG GGGATGTGCT GCAAGGCGAT

3781 TAAGTTGGGT AACGCCAGGG TTTTCCCAGT CACGACGTTG TAAAACGACG GCCAGTGAAT

3841 TGTAATACGA CTCACTATAG GGCGAATTGG GCCCTCTAGA TGCATGCTCG A**GCGGCCGC**T

3901 GCACAAACAG ATATTTGGAA TGAATATATA ACCAAATAAA GACATATTTG GATTTCATAT

3961 CCTTACACTT GTCTCAGAGA TATCACAATA CAGAAAAATA AATTTATGCT TAAAATAGTG

4021 ACTTAAAAAG AACAAAGATA TACTCAACTT CAAAAGGCAA GAGTAAAAAG AATATTCTGG

4081 AAATGTTATT GATTCTGTAA CAAAATAAAT AATTTATTAA TTTATTAAAT AAATAATTTG

4141 TGTTTTCCAA AGCCAACTTA TAAAAATTAA ATAATTACTT GTTGATAAAA TATTAATTGA

4201 AATATTCTTT TTTCTAAAAC CCTTGGTAAT CTTTGTGTAT TGAGAGTAGA ATGAATCAGT

4261 ATGCATGTTA TTGGAATACA GTGTGGTATG AAGCACTACA TGACCAGCAT GCACTGATCA

4321 GTAATTAGCA TTTTCTTTTC CTAATAATTT CTTTATGGTT TAAGCCTTTG AGGTCCTCTC

4381 TCCAAGTACA TAAAACATAT ATAGGTGATG TGAACTATTA TTATCCTATT GTTCTGTGAA

4441 GCACCAAAAT TTTATTTCCT CTATCTAATT GTGTTTTGGT ATATGCTATC TAACATTCCC

4501 ATTCCTGCCT AGCTTCTAGG AATCAGTATT CGGCATTCAA ATTAATATTT ATAGCTTCTT

4561 CTATAGTTGA CTTATTTCAC TTAACAGGAT TTCGCTTTTT AAAACATTTT TATTAGATAT

4621 ATTCTTTATA TACATTTCAA ATGCTATCCC GAAAGTTACC TATACCCTCC CCCTGCCCTG

4681 CTCCCCTACC CACCCATTGC CACTTCTTGG CCCTGGCATT CCCTTGTACT GGGGCATATA

4741 AAGTTTGCAA TACCAAGGGG CCTCTCTTCC CAGTGATGGC CGACTAGCCC ATCTTCTGCA

4801 TATGTTGCAG ACCCTTTCAG CTCCTTGGGT ACTTTCTCTA GCTTCTCCAT TGTGGGCCCT

4861 TTGTTCCATC TTATAGATTA CTGTGAGCAT CAACTTCTGT ATTTGCCAGG CACTGGCATA

4921 GCCTCAAACG AGACAGCTAT ACCAGGGTCC CTTCAGCAAT ATCTTACTGG CATATGCAAT

4981 AGTGTCTGTG TTTGGTGGCT GATTATGGGA TGGATCCCCG GGTGACTGCC TTTTAGTTCC

5041 ATTCTCTTTT TCTGTTGTAA ATGGCATGAT TTCACTCTTC ATTATGATAC ATTGTGTACA

5101 TATACTTTAT TAATCCATTC ATCTGTTGAT AAACATCTAT GCTGCCTCTA TATCATGAGC

5161 ATTATAAATA GTGCCACAAA AACACCTGGA AGTGCAGTTG TATCTTTGAA TTAATGAAAT

5221 TATTTTCTTT GGATATATGC ACAGGTGTGT AAATATGGAT CACAGAAAAG GTCCATTTTC

5281 AGTGTTCTTT CCACCCTTTA CAGACTTTGC TGCTTCTTAT CCCCTTTCAA CTTTACTTCT

5341 GATATATATG TTTCAGCAAT AAACATATTC ACAAATAAAT GCGAACCTTG GACACATTGA

5401 ATTTTTAGAG ACATATTCTG TGTTCACTTT CCTGTGTACA TTTGACTTAT TATGTCCTTT

5461 TACTACTACT CTCCCAAACT GAATGATGTA CCTTTCATAA TGACGTTCAG CTGTCCTTAC

5521 TAATGTATTC TCAAAGATAA TATTAGAACT TAGACCACAT TAGATCTTAC TATTAGCATC

5581 CTTTAAATAC TTGTCACTTT CAGGGGCATA GAGGTGTGGA ATGGTCAATG AAGAGCTTTC

5641 AATTCCCCTG CCTACATCTC TCACCTAATT TTTATACCCA TCTTCTTAGG TAGATTGTAT

5701 TATCATCCCT GTCCTACACA TAAGGAAATT GTGGTGAATC CGGAGTCAGA ATTAACATTG

5761 CCTAAGTTCG ATATTGTCAT GAAAATTATG CATTACACAA TTTAATAATG TTCCTGTATT

5821 ATCAATTTGA AGAATCTGTA GAAAGTATGT ATATATGTAG CTAAAGAAAA GTGTGCATAT

5881 TTATATAAAT GTATAAACTC AGTTTTTCAA AAGATCCAAC TCTATCACCA GTTACTGCAG

5941 TAATTCAGTA TTGACGCAAA ACCTAGTAGT TCCTGTCAAT AATTATAAAT TTATTTCTTA

6001 AATGATGAAA TTGCAGAATA AAGTAAAACC AACCCATGCT TTATTACAGT AAAGAGGTAA

6061 CAATACATGT AAACTGAGTA GCTAAATCAG AGGTCCCTGC AGGTGAACTG ACTTGGTCAA

6121 TGTTGCTTAA ACAAAGCAGA GCCTGCTGAA AACATGGGGC AATTCTTTGA AATGTGAAGG

6181 GAATTTCAGT TTGCAATATA AGAATCTGGC ATTGGTATGG TCTAGAAAAG ACTTGAGCAG

6241 TGGTTCTCCG TCTTTCTAAT GCTGAGACCC TCTAATATGT TTCCCCATGC TGTGGTAACC

6301 CCCAACCATA AAATTATTTT TGTTGTTATG TCATAACTGT AATTTTGCTG CTGATATGAA

6361 ATGTAATGTA AATATCTGTG TTTTCCAATG GTCTTAGGTG ACCTGTGTCA AAGGGTCATT

6421 TGACCTCCCC AACGGGTCAT GACCGACCAA CAGTTTGGGA ACGACTGGAT TAGAGAGAAG

6481 CCAAATAGCT CTGTAGTAAA ATCAGGGTGA TATTTCCTGT AGCACGCTTG TAAGTATTCA

6541 AGTAGATTTT CAAGCACATT GTATGGTATA TGACATTTGT TAACTTTGTC TTTTATGGCA

6601 TTAGAGTAAG TGTTGCTACT GTAATAGTCT TGAGGTCGTG GCTGTTTGCA TGACAGGTGG

6661 TTTTCTAGAC CAGAGTGGTA ATGGAGGACA GAAGGGACAG AGAAAATATC TGTGTCAGAA

6721 GCAGAGAAAG AACACCAGCG ATTGTGGAAT TTTGGGCGAA CTTTGGATTT TTACAAGAAT

6781 GGGGATTCAA TCCATGCAGC TTGATATGAA AAGTCTGGAG AACCTTAAGT GACTGTTTTG

6841 AGTACACTGG AGAAACTTGA CCATTCACTT AAAAAAAAAA AAAAGTAACG TTAATTGCTA

6901 GAACTGAGAC GTCGACAATG GCATAGCACT TTCTTAAAAA TGAATTCAAA TATATCAGCA

6961 TATGCAGTCA ACTAATTTGC TGACTTGAAG ACAGCTTCCC AAACCTATTA TTGCCTGTAA

7021 TGAACGACTA TATGCACTAA TCACAACATG CAGTCTTGAC AGTGCTCACG GCATCTAAAA

7081 AAATCATGAT ACAGGGATTT TTCTTTAGAC TCAACACATT TTTCATTCAG AAACCTGATT

7141 GGCTGTTCAA AGTGCCCTGC ATGATATAAA TTGGGGCACA GAGTTGGAAG AAACCATCGG

7201 ATCAAGCATC CCTGAGCTTC AGACAGAAAC TCACTGAGCA TACACTCAAA GGTATGTGGA

7261 TTTCATTCAT GATTTACCAT CTCCACTGTG GTCATTTCTT TCTCTGAATT TCATGTTTCA

7321 AATGTGTGAA TGAATTGCAC ATTTCTAATT CTTTGTGCCA TCTACACCAT ATGTACTTCT

7381 TTAGTTTTAC TATAATGAAA TTATAGACTT AAATCATTGA AAAGAAGTTT TAACTTTATA

7441 GTACAGTAAT GTATGTACTT CTGTAAATTT TAGGTCAAGC ATTCCAAAGC CTTTTGGGAG

7501 CCAGATTTGA TACAAAAATG TGTATTTGCT TTTTAGTTAA GATGCTGTTT TAACAGGCTG

7561 AGGGCTACTT CTTTACAAAA TCTTGTTAAA TCTTGTGAAT CCTTTTTCTT ATTACCTCAA

7621 CCTTTGGGGG GAAAAGGTTT AGATGTTAGA AATCCTCAAT GTATTTTTCA TAGGTAACAC

7681 CAATGCATTT TACATTCATG TCCTAAATTT ACTTTCAAGG TGTTGTCCAA AACCAATGGC

7741 ATATTTTAGG ATAGGTAGTT CTTGCTTCAT GTTTCAAAGG CCTTAATATG TAGAAGGAAT

7801 ACAGTATAGT TGACAAGCAA GAAGAGGGTG CTACTGGCTT AAGACAGAAC TCTTTACGTA

7861 GAAACCATTC CACAACCCTT AAAGAAGCTT AGAATGGTAG CACTATTCTC TTTCTTTGGA

7921 TTAAGCATGC CTTCTGCTTC AAACCTGGAG TTTAAAGGCA TTATGAGAGA TTCTTGCCTA

7981 AAATTCTACA AGTGGACCTA AACCCACTCT GCCATTAATC CATTCTGTAA AATTATTCAA

8041 TATTTTCTCA AATAATTACT CTATGTATTT AACAGTAGCA ATGCTCAAGT GGGTTATGCC

8101 CCCCAAAAGA AAAACAACAT TTCTATCTTT TCTTGTTACC AAAGTATAAA ACCATGTTCA

8161 TTTGATTATC TCTCCAACTG TTATACTCAA CCCGATTCTT TTAATCAGTT TTATTGTTTC

8221 CATCTAGCTT CTAGGCATCC TTAGGCTGTA TAGCATAGAA AATGGTTTGC AGCATCATTC

8281 TTGTAGATCA CTTTTGGTCC TCTAACTCGT TAACCTTCAG CTTCAGCTTA GTTTTTAAAC

8341 AAATAAACAC ATTGCAATAT TAGTACTCCA GAAGCAATGA TAATTCCATA TGAATGTCAC

8401 TTTACAATAA GGGCCGCACC TTCTTTTTGA TTAGCAAGAC ATTAATGTGG ATTATATACA

8461 TGTATGAAGC ATAATTATAT TCATGCTTTT AAAAGTTTTT AAATGGTTGG TATTGATTTA

8521 CATTTCAGAA CCATCAAG**GG CGCGCC**GAAA AC**ATG**TTGCT GCTTCAGTGC AGAAATCCGA

8581 CTTCTCCTCC AAAGCCATGT GGCCTGGTAC CAAATGTAAA GATGAGTCTC CTTGTTTTCC

8641 TGGGTCTGCT TGGTGTCTCT GCTGCCATGC CATTCCAGAT GCCAATGCCC CGAATGCCTG

8701 GATTTAGCAG TAAAAGTGAA GAGATGATGC GATATAATCA ATTCAACTTC ATGAATGCCC

8761 CACCAATGAT GCCTATGGGC CCATATGGAA ATGGTATGCC AATGCCGCCA CACATGCCTC

8821 CACAGTACCC TCCATACCAG ATGCCCATGT GGCCTCCACC AGTACCCAAT GGATGGCAGC

8881 AACCCCCAAT GCCCAATTTC CCAAGCAAGA CTGATCAAAC CCAGGAGACC GCCAAACCCA

8941 ACCAGACCAA TCCACAAGAG CCACAGCCAC AAAAGCAGCC TTTAAAGGAA CCACCAAATG

9001 AAGCAGCACG GGCCAAAGAT GACGCCCAGC CACCTCAGCC ATTCCCACCA TTTGGCAATG

9061 GACTTTACCC CTATCCACAA CCACCATGGC CAATTCCACA GAGGGGACCA CCAACAGCGT

9121 TTGGACGGCC AAAGTTCAGC AATGAAGAAG GAAATCCTTA CTATGCATTT TTTGGATATC

9181 ACGGCTTTGG GGGTCGTCCT TATTACTCAG AAGAGATGTT TGAAGATTAT GAAAAACCCA

9241 AAGAAAAAGA CCCTCCTAAA CCAGAGGACC CACCTCCAGA TGACCCACCC CCAGAGGCCT

9301 CTACAAACTC AACTGTGCCT GATGCTAATG CCACTCAATC AATTCCTGAA GGCGGAAATG

9361 ACACTAGCCC AATAGGAAAC ACAGGCCCTG GGCCGAACGC TGGGAACAAT CCTACAGTTC

9421 AAAACGGTGT CTTCCCTCCC CCTAAAGTTA ATGTTTCAGG CCAGGGAGTA CCAAAAAGCC

9481 AAATTCCGTG GAGACCAAGT CAGCCAAATA TTTATGAGAA TTATCCTTAC CCAAATTATC

9541 CTTCAGAAAG ACAATGGCAA ACCACTGGTA CCCAGGGGCC TAGACAGAAT GGACCTGGCT

9601 ACCGAAATCC ACAAGTTGAA AGGGGTCCTC AGTGGAATTC CTTTGCTTGG GAAGGCAAAC

9661 AAGCTACTCG TCCAGGAAAC CCAACTTACG GTAAACCTCC CTCTCCTACC TCCGGGGTTA

9721 ATTATGCAGG AAATCCAGTC CATTTCGGAA GAAACCTGCC AGGGCCAAAT AAACCCTTTG

9781 TGGGAGCCAA TCCGGCCTCA AATAAACCCT TTGTGGGAGC CAATCCGGCC TCAAACAAAC

9841 CCTTTGTGGG AGCTAATCCG GCCTCAAACA AACCCTTTGT GGGAGCCAAT CCGGCCTCAA

9901 ATAAACCCTT TGTGGGAGCC AATCCGGCCT CAAATAAACC CTATGTGGGA GCCAATCCGG

9961 CCTCAAACAA ACCCTTTATA GGAGCCAATC CGGCTGCAAA CAAACCATCT ATAGGAACCA

10021 ATCCAGCCGC AAACAAACCA TCTATAGGAA CCAATCCAGC TGCAAATAAA CCCTTTGTGA

10081 GAAACAATGT AGGTGCAAAT AAACCCTTTG TGGGAACCAA TCCCTCCTCA AACCAACCAT

10141 TTCTGAGAAG CAATCAGGCC TCAAATAAAC CATTTATGAG AAGCAATCAG GCCTCAAATA

10201 AACCATTTGT GGGCACCAAT GTGGCCTCAG TGGGTCCTAA ACAGGTCACT GTTAGCCACA

10261 ATATGAAAAC TCAAAATCCA AAAGAAAAGT CACTAGGTCA AAAAGAAAGA ACAGTCACTC

10321 CTACCAAAGA TGCAAGCAAC CCCTGGAGAA GTGCTAAACA ATATGGAATT AACAATCCAA

10381 ACTATAATTT GCCTCGCTCT GAGGGCAGCA TGGTAGGCCC AAATTTTAAT TCCTTTGATC

10441 AACAAGAAAA CTCCTACTTC TCAAAAGGAG CTTCCAAAAG AGTACCAAGT CCTAATATAC

10501 AAATCCAAAG CCAGAATTTG CCCAAAGGAA TTGCTTTAGA GCCAAGAAGA ACCCCATTTC

10561 AATCAGAAAC TAAAAAACCT GAGTTAAAAC ATGGTACACA CCAGCCTGCA TACCCTAAGA

10621 AAATCCCTTC TCCTACAAGA AAACATTTCC CTGCTGAAAG AAATACCTGG AATCGTCAAA

10681 AAATCCTTCC ACCCTTAAAG GAAGACTATG GGAGGCAAGA CGAAAATTTA CGTCATCCGT

10741 CCTATGGCTC TAGAGGAAAT ATTTTTTACC ATGAATATAC CAATCCTTAT CATAATGAAA

10801 AATCACAGTA CATTAAAAGC AATCCATGGG ATAAGAGCTC TCCCAGTACT ATGATGCGGC

10861 CAGAAAACCC ACAGTACACC ATGACTTCTC TAGACCAGAA GGAGACAGAG CAGTACAATG

10921 AAGAGGATCC AATTGATCCA AATGAAGATG AATCTTTTCC AGGACAAAGT AGATGGGGGG

10981 ATGAAGAGAT GAACTTCAAA GGAAACCCAA CAGTTAGGCA GTATGAAGGT GAGCACTACG

11041 CCTCAACCCT AGCGAAGGAA TACCTTCCTT ATTCCTTAAG TAATCCACCA AAACCCAGTG

11101 AAGATTTTCC TTACAGTGAA TTCTATCCCT GGAACCCACA GGAAACGTTT CCAATATATA

11161 ACCCAGGTCC TACTATAGCA CCACCCGTGG ACCCCAGAAG TTATTATGTT AATAATGCCA

11221 TAGGACAAGA AGAAAGCACT CTCTTTCCTT CATGGACCTC CTGGGACCAC AGGAATCAAG

11281 CTGAGAGGCA GAAAGAGAGT GAGCCATATT TTAACAGAAA TGTCTGGGAT CAGTCAATAA

11341 ATTTACACAA ATCTAATATA CCAAACCATC CTTATTCCAC TACATCCCCT GCTAGATTTC

11401 CAAAAGATCC AACATGGTTT GAAGGTGAGA ATTTGAACTA TGATTTGCAA ATTACTAGTT

11461 TAAGTCCACC AGAAAGAGAA CAGTTGGCTT TCCCAGACTT CCTGCCTCAA AGTTACCCAA

11521 CAGGTCAAAA TGAAGCACAC TTATTTCACC AAAGTCAAAG AGGGTCTTGC TGTATTGGTG

11581 GCTCCACAGG ACATAAAGAC AATGTGCTGG CTCTACAAGA CTACACTTCA TCCTATGGTC

11641 TTCCACCAAG GAAGAACCAA GAAACCAGTC CAGTGCATAC AGAAAGCAGT TATATCAAGT

11701 ATGCAAGACC TAACGTTTCC CCAGCAAGCA TCCTACCTAG TCAAAGAAAT ATCTCAGAGA

11761 ACAAACTAAC TGCAGAAAGC CCAAACCCAA GTCCATTTGG AGATGGTGTG CCTACTGTGA

11821 GGAAAAATAC TCCATATTCT GGAAAGAATC AACTAGAGAC AGGAATTGTG GCCTTTTCTG

11881 AAGCCAGCTC TTCTCAGCCA AAAAACACGC CCTGTCTTAA AAGTGACCTT GGAGGAGATC

11941 GGAGGGATGT TCTGAAACAA TTTTTTGAAG GCAGCCAGCT GAGTGAAAGA ACTGCTGGCC

12001 TTACTCCTGA GCAGCTCGTC ATTGGTATTC CTGATAAAGG CTCTGGCCCA GATAGCATAC

12061 AAAGTGAAGT CCAAGGAAAA GAGGGTGAGA TGCAGCAACA AAGGCCACCT ACCATCATGA

12121 AGTTGCCATG CTTTGGCTCC AATTCAAAAT TTCACTCTTC TACCACTGGA CCTCCAATTA

12181 ACAACAGAAG ACCAACCCTA CTTAATGGTG CTCTCTCCAC ACCCACTGAA AGTCCTAACA

12241 CATTGGTTGG GTTAGCTACT AGGGAACAAC TTAAAAGTAT AAACGTAGAT AAACTTAATG

12301 CAGATGAACA CACTACACTC GAATCTTTTC AAGGAACCAG TCCACAGGAC CAAGGCTGCT

12361 TACTGCTTCA GGCT**TAG**GGA TCGCTTCAAC T**GCGATCGC**C GAAGTGGATA CTTTGGTTGT

12421 TTTTAGGAAT AACTCAAGAA CACAATGATT TGTGCCTACA ATCACTTAGT AAATTCTGTA

12481 ACTAAAAATA AGTATCATTA GCAGATAATA AAATGTTTGA AAAATCATTC ATGTCTTTGT

12541 GTTGAATTAA ATTTAAATTT TCCTATCACT TGAGAGAAGA ACTTATAAGT GAATAATATA

12601 GGGACTGGTC ATTTGCTCCT ACAAATTGAC CATTTAGCAG GCAAATCATG ACTATATCTT

12661 CTTAGAGAAC TTTTATATTA TCTGTAATGT TTTCTTAAAA TAGAAATTTG TACATCATAA

12721 ACAATAGCTG AGAAAGTTAA CAGAAATAGT TAAGCATGGA TAGTCACTGA ACCTGAACCA

12781 AATGAAGGCA AGTTTAGAAG AAATATTCTT TACATTGAAA AAGATAAACT TTAAGGAATG

12841 TTTATATTCT AAATCAAATT AAGACTTGGA ACTTTAGCTT TATTCTTCAC ACCACATCAG

12901 TAACCAAAAT CTGGTCTTTT GATCTCATTC TATAGAAAAA TATTTTGAGT GAAATTATGT

12961 GATATATATG GTAATTCACT GAAATTTAAA TTATTAAAAT AATTATTTTT CCTAAGAAAA

13021 TTAATAACGG AGCCTCAAAC AGAGAATTGC AGTGATTTTA TACACAAGTC ATTTCTTTCA

13081 CCTCATAATC TTTAAGCTGA TTAAGTCTAC CACATGGTCT TCAAAACGCT AACAGATGCG

13141 TAAAATGGAC TCAAAACAAG TTTTCTTTCC CACTCATATT TATATTTGAA TCATTTTTCC

13201 TCTTACACCC ACACAAAATT GTAAACAGTC TTTGCAGGAT TTATGTTACA CAGCACCCAC

13261 AGTAACTTTA TTCCAGTACC TTCATGATTA AGGTCTCTCT TAATCATTTT CTGGAATGAA

13321 TAAACATAGC TTTCTCTGAG AAGATATCAA TATGCAAACA AATTAGTTGA TTTTAAAGAA

13381 CTAAAATGCA GTAATAAAGG TAAAAATAAA GCATTTAATG AATTAGCATA GGCCACTTCT

13441 AATCAACTGA GTAAATATTT CTCAAGCACT CACTAGACTG GGTATTACTG TATGTGATAG

13501 TGAAAACAGT AATTCCTTAT CCTTAG**GCCC GGGC**
